# Micro-Players of Great Significance—Host microRNA Signature in Viral Infections in Humans and Animals

**DOI:** 10.3390/ijms231810536

**Published:** 2022-09-11

**Authors:** Ewa Ostrycharz, Beata Hukowska-Szematowicz

**Affiliations:** 1 Institute of Biology, University of Szczecin, 71-412 Szczecin, Poland; 2 Doctoral School, University of Szczecin, 71-412 Szczecin, Poland; 3 Molecular Biology and Biotechnology Center, University of Szczecin, 71-412 Szczecin, Poland

**Keywords:** microRNA, viral infections, hepatitis viruses, hemorrhagic viruses, respiratory viruses, *Lagovirus europaeus*/RHDV, Marek’s disease virus, foot-and-mouth disease virus, porcine reproductive and respiratory syndrome virus, rabies virus

## Abstract

Over time, more and more is becoming known about micro-players of great significance. This is particularly the case for microRNAs (miRNAs; miR), which have been found to participate in the regulation of many physiological and pathological processes in both humans and animals. One such process is viral infection in humans and animals, in which the host miRNAs—alone or in conjunction with the virus—interact on two levels: viruses may regulate the host’s miRNAs to evade its immune system, while the host miRNAs can play anti- or pro-viral roles. The purpose of this comprehensive review is to present the key miRNAs involved in viral infections in humans and animals. We summarize the data in the available literature, indicating that the signature miRNAs in human viral infections mainly include 12 miRNAs (i.e., miR-155, miR-223, miR-146a, miR-122, miR-125b, miR-132, miR-34a, miR -21, miR-16, miR-181 family, let-7 family, and miR-10a), while 10 miRNAs are commonly found in animals (i.e., miR-155, miR-223, miR-146a, miR-145, miR-21, miR-15a/miR-16 cluster, miR-181 family, let-7 family, and miR-122) in this context. Knowledge of which miRNAs are involved in different viral infections and the biological functions that they play can help in understanding the pathogenesis of viral diseases, facilitating the future development of therapeutic agents for both humans and animals.

## 1. Introduction

MicroRNAs (miRNAs, miR) are small (i.e., consisting of 17–25 nucleotides) non-coding RNA molecules involved in the regulation of gene expression [1,2]. MiRNAs are found in most eukaryotes, including humans and animals (referred to as host miRNAs in the native organism) [2]. MiRNAs are critical for correct development in all organisms. They also play a crucial role in regulating gene expression, as well as controlling cell differentiation, apoptosis, and metabolic pathways, including homeostasis [3,4]. In the human genome, miRNAs account for 1–5% and regulate at least 30% of protein-coding genes, where the aberrant expression of miRNAs has been found to be associated with many human diseases [5,6]. Furthermore, miRNAs can also be secreted into extracellular biological fluids, where they may serve as potential biomarkers [7]. MiRNAs are also involved in the regulation of the immune system and response, including the response to viral infections in humans [8,9] and animals [10,11].

**Figure 1:**
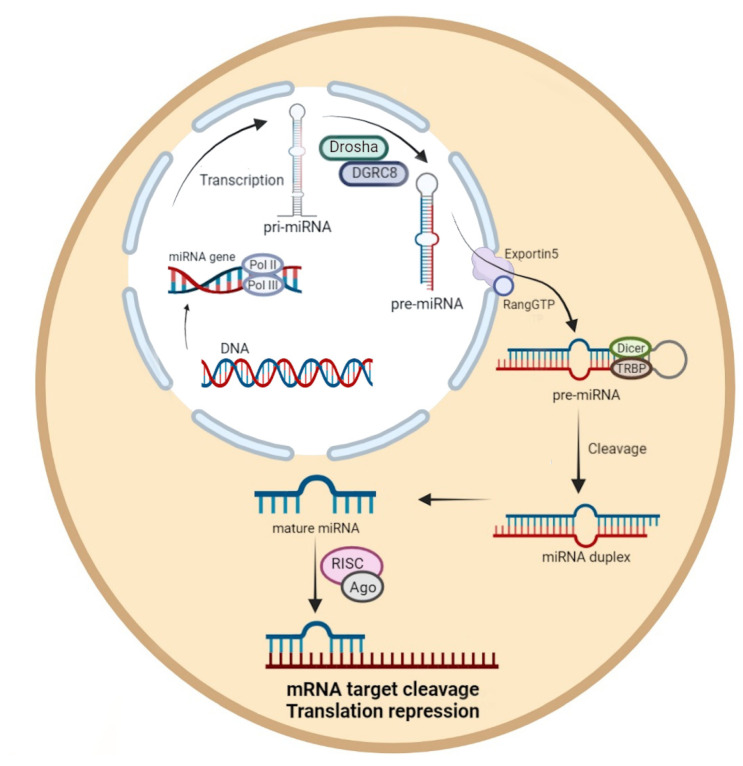
Biogenesis and functions of microRNA in humans and animals. MiRNA genes are transcribed in the cell nucleus to pri-miRNA, then, transformed to pre-miRNA by Drosha. Then, the pre-miRNA is transported to the cytoplasm by Exportin5 and cleaved to create an miRNA duplex containing the mature miRNA. After strand separation, the single-stranded mature miRNAs are incorporated into the miRNA-induced silencing complex. The resulting complex mediates the recognition of target mRNA and participates in gene silencing via translation repression or mRNA cleavage.

MiRNA precursors are commonly located within the genome. As such, miRNA biogenesis initiates in the nucleus, and involves a two-step process [2] (Figure 1). The first step of miRNA formation is gene transcription by Pol II or Pol III in order to generate primary miRNA (pri-miRNA). The pri-miRNA maturation process begins with nuclear cleavage into stem-loop structures (70 nucleotides on average), thus forming precursor miRNA (pre-miRNA) [12]. This process is accomplished by a protein complex consisting of the RNase III endonucleases Drosha and DiGeorge syndrome critical region gene 8 (DGCR8), which control miRNA biogenesis through stabilization by Drosha. Afterwards, the pre-miRNA is exported to the cytoplasm in an Exportin5/RangGTP-dependent manner, and cleaved near the loop by Dicer and an RNase III to form an miRNA duplex containing the mature miRNA [13]. The duplex unwinds to produce single-stranded mature miRNA, which assembles into a ribonucleoprotein complex (RNA-induced silencing complex; RISC) which contains the Argonaute (Ago) protein. The resulting complex mediates the recognition of target mRNA and participates in gene silencing via translation repression or mRNA cleavage [14]. Most miRNAs in animals are thought to function through the inhibition of the effective mRNA translation of target genes via imperfect base pairing with the 3′-untranslated region (3′UTR) of target mRNAs [2].

### MicroRNAs and Viral Infections in Humans and Animals

MiRNAs have been also shown to play an important role in viral infections in humans and animals, and can therefore be considered micro-players of great significance. MiRNAs in humans and animals are referred to as host miRNAs [2]. In recent years, it has been shown that some viruses can also synthesize their own miRNAs [15]. According to the above, Barbu et al. [11] has classified miRNAs based on their source and role. With respect to their source, they were classified into host miRNAs and viral miRNAs. However, due to their various roles, the host miRNAs were further divided into (i) pro-viral miRNAs and (ii) anti-viral miRNAs. Considering the former, the interaction between viruses and host miRNAs may facilitate viral replication and infection, thus exerting a pro-viral function. Furthermore, pro-viral miRNAs can promote viral infection by suppressing anti-viral factors, such as interferon (IFN), thus allowing the virus to escape the immune response of the host. In contrast, various host miRNAs can exert anti-viral functions by influencing the production of viral RNA, blocking viral replication, suppressing pro-viral proteins, or inducing the virus to enter a latent phase [11,16].

The purpose of this comprehensive review is to present the key miRNAs involved in the course of viral infections in humans and animals. Infections caused by viruses constantly threaten the lives of humans and animals, constituting a global health problem. In the case of humans, it is safe to say that they pose a significant challenge for modern medicine; meanwhile, in the case of animals, they may lead to huge economic losses, requiring effective and rapid diagnostic and therapeutic pathways. Knowledge of which miRNAs are involved in different viral infections and their biological functions can help to understand the pathogenesis of viral diseases, as well as facilitating the future development of therapeutic agents for both humans and animals.

## 2. MicroRNA Signature in Human Viral Infections

**Figure 2:**
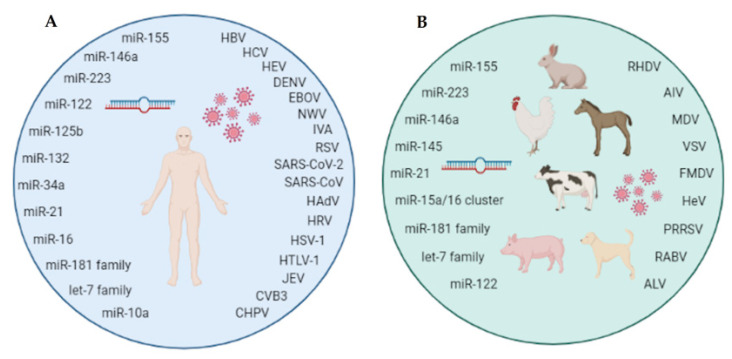
Key miRNAs: (**A**) in human viral infections and (**B**) in animal viral infections.

In the scope of this study, we distinguished 12 host miRNAs which are extremely important, from the point of view of their participation in viral infections (Figure 2A).

### 2.1. MicroRNA-155 (miR-155)

MiR-155 is a multi-functional miRNA which is highly expressed in several immune cells [17]. The expression of miR-155 was first reported in human spleen and thymus, liver, lung, and kidney [18]. MiR-155 is particularly responsive to many inflammatory stimuli, such as tumor necrosis factor alpha (TNF-α), interleukin (IL)-1β, IFN, pathogen-associated molecular patterns (PAMPs) and damage-associated molecular patterns (DAMPs), alarmins (e.g., IL-1α), and hypoxia, as well as the Toll-like receptor (TLR) ligand in various cell types, particularly in monocytes/macrophages [19]. MiR-155 has an important function in the modulation of humoral and cellular immune responses during viral infections, and can be inhibited by viruses [20].

#### 2.1.1. Hepatitis Viruses

Hepatitis B virus (HBV) belongs to the family *Hepadnaviridae*, with a DNA genome [21]. HBV, as an etiological agent of viral hepatitis, is a public health concern. Chronic HBV infection leads to persistent liver inflammation and damage, which may ultimately result in hepatocellular carcinoma (HCC) due to the development of oncogenic changes [21]. The expression level of miR-155 is significantly down-regulated in healthy hepatocytes; however, it may be increased in different pathological processes. Due to its role in regulating the immune response, miR-155 plays a significant role in infections with hepatotropic viruses, such as HBV or hepatitis C virus (HCV) [22]. Su et al. [23] ] performed a study to identify the effects of miR-155 on the immune response during HBV infection in human hepatoma cells. They found that the ectopic expression of miR-155 up-regulated the expression of several IFN-inducible anti-viral genes. This study also showed that enhanced expression of miR-155 suppressed the suppressor of cytokine signaling 1 (SOCS1) and enhanced the phosphorylation of signal transducer and activator of transcription (STAT) 1 and STAT3. In addition, miR-155 may inhibit HBV X gene expression, to some extent, in vitro. They also showed that miR-155 can promote intrinsic anti-viral immunity by targeting SOCS1, thus enhancing the Janus kinase/signal transducer and activator of transcription (JAK/STAT) signaling pathway, which suppresses HBV infection in hepatocytes [23]. In a liver biopsy specimen, serum and peripheral blood mononuclear cells (PBMCs) from HBV-infected patients were observed to significantly reduce miR-155 [24,25,26]; however, interestingly, chronic hepatitis B (CHB) patients with elevated alanine aminotransferase (ALT) presented higher levels of miR-155 [25,26]. These results suggest that miR-155 levels in PBMCs are correlated with immune state in patients with chronic HBV infection [25]. Furthermore, the down-regulation of miR-155 in the natural killer (NK) cells of immune-active patients impaired IFN-γ production by targeting SOCS1, which may contribute to immune dysfunction [26]. The miR-155 level was also reduced in hepatoma cells (HepG2.2.15) stably replicating HBV. Furthermore, due to the fact that miR-155 has heterogenous roles in the immune responses mediated by TLRs, Sarkar et al. [24] investigated the relationship between TLRs and miR-155, and the consequent effect on the replication status of HBV. They observed a positive correlation between TLR7 and miR-155 expression, which modulated HBV replication. In addition, their results indicated that TLR7 stimulation induces the synthesis of miR-155 through the NF-κB pathway [24]. Meanwhile, the ectopic expression of miR-155 in HepG2.2.15 cells reduced viral DNA via targeted suppression of CCAAT/enhancer-binding protein-β (C/EBP-β), which is a positive regulator of viral transcription [24]. During CHB and HBV infection in carriers, higher miR-155 expression in CD4+ and CD8+ T-cells has been observed, which was positively correlated with T-cell activation. In addition, HBV carriers expressed higher amounts of miR-155 in their CD8+ T-cells compared to healthy individuals [27]. Wang et al. [28] reported that hepatitis B antigen (HBeAg) augmented the expression of miR-155 in macrophages through the phosphatidylinositol 3-kinase (PI3K) and nuclear factor kappa-light-chain-enhancer of activated B-cells’ (NF-κB) signal pathway, where the increase in miR-155 promoted HBeAg-induced inflammatory cytokine production by inhibiting the expression of B-cell lymphoma 6 (BCL-6), inositol polyphosphate 5-phosphatase 1 (SHIP-1), and SOCS-1. An interesting observation, made by Chen et al. [29], is that miR-155 was inhibited by HBV infection, while miR-155 transfection remarkably reinforced HBV replication and antigen expression in HepG2.2.15. Moreover, miR-155 impaired the inhibition of the SOCS1/protein kinase B/mammalian target of the rapamycin kinase (SOCS1/Akt/mTOR) axis and reinforced autophagy, resulting in increased replication of HBV [29]. Another interesting fact is that over-expression of miR-155 in serum was correlated with non-responsiveness to the hepatitis B (HB) vaccine. Researchers have hypothesized that increased miR-155 expression may dampen the generation of the T-cell-mediated immune response, resulting in failure to respond to the HB vaccine [30]. Furthermore, rs767649 T/A polymorphisms in miR-155 may be a risk factor for CHB. It has been shown that AT and AA genotypes are related to the risk of developing chronic hepatitis. Polymorphism of miR-155 can also affect the binding sites of interferon regulatory factor 1 (IRF1), interferon regulatory factor 2 (IRF2), and PR domain zinc finger protein 1 (PRDM1), thus playing a part in the pathogenesis of CHB [31].

Hepatitis C virus (HCV) belongs to the family *Flaviviridae*, with the ssRNA genome. HCV, as opposed to HBV, causes chronic viral hepatitis in around 55–80% of infected individuals. Around 5–20% of chronic hepatitis C (CHC) patients develop cirrhosis or HCC [32]. In patients with CHC infection, the expression of miR-155 has been found to be elevated in serum, monocytes, and liver tissue [33,34]. The stimulation of normal monocytes with TLR4 and TLR8 ligands or the HCV core, non-structural protein 3 (NS3), and non-structural protein 5 (NS5) recombinant proteins induced a robust increase in both miR-155 expression and TNFα production, thus identifying potential mechanisms for the in vivo induction of miR-155 during chronic HCV infection [33]. The up-regulation of miR-155 expression was also observed in PBMCs from HCV-infected patients, and was associated with HCV replication in PBMCs cells [35]. Similarly to HBV infection, miR-155 was down-regulated in NK cells from chronically HCV-infected individuals. Lower levels of miR-155 were correlated with up-regulated T-cell immunoglobulin mucin-3 (TIM-3) and T-box expression in T-cells (T-bet) and NK cells during chronic HCV infection. The transfection of miR-155 to NK cells affected T-bet/Tim-3 expression and increased in IFN-γ production. From the above, it was concluded that miR-155 may regulate Tim-3/T-bet/STAT-5 signaling and cytokine expression in NK cells, potentially balancing immune clearance and immune injury during chronic viral infection [36]. In non-parenchymal liver cells (NPCs), miR-155 expression was induced by the TLR3 ligand, while transforming growth factor beta (TGF-β) and IL-10 inhibited the TLR3-induced anti-viral response through the inhibition of NF-κB and IRF3. However, in liver biopsies from patients infected with HCV, a lower level of miR-155 expression was associated with higher expression of interferon-stimulated gene 15 (ISG15) and TLR3 [34]. In contrast to other genotypes of HCV, investigation of the HCV genotype 4 indicated that the expression of miR-155 was similar in both healthy and infected PBMCs [37,38]. This could be attributed to the attenuation of the IFN pathway by HCV, which was assessed by investigating the expression of an ISG, which showed lower expression in HCV-infected PBMCs. HCV might also interfere with miR-155 expression through the TLR-7 pathway [38]. Moreover, miR-155 also plays an important role in regulating tumorigenesis; in particular, HCV infection may promote the initiation and progression of HCC. Zhang et al. [39] indicated that miR-155 levels were markedly increased in patients infected with HCV. MiR-155 transcription was found to be regulated by NF-κB, where p300 increased NF-κB-dependent miR-155 expression. The over-expression of miR-155 significantly inhibited hepatocyte apoptosis and promoted cell proliferation by repressing the adenomatous polyposis coli (APC) gene and activating wingless-related integration site (Wnt) signaling. The up-regulation of miR-155 and activation of the Wnt pathway resulted in nuclear accumulation of β-catenin and a concomitant increase in cyclin D1, MYC Proto-Oncogene (c-Myc), and survivin, contributing to HCC growth [39].

#### 2.1.2. Hemorrhagic Viruses

Dengue virus (DENV) is a member of the *Flaviviviridae* family, with an ssRNA genome, and includes four serotypes (DENV-1, DENV-2, DENV-3, and DENV-4), each of which is capable of causing dengue fever (DF) and dengue hemorrhagic fever (DHF)/dengue shock syndrome (DSS) [40]. MiR-155 also plays a significant role in DENV infection, and has been determined to have a major function in regulating the TLR/NF-κB-induced signaling pathways in human macrophages in DENV infection [41,42]. Su et al. [43] indicated that miR-155 is down-regulated in DENV-infected Huh-7 cells and infected mice. However, the exogenous over-expression of miR-155 appeared to limit viral replication in vitro, suggesting that low miR-155 levels would be beneficial for DENV replication. In vivo, the over-expression of miR-155 protected mice from the life-threatening effects of DENV infection and reduced the propagation of the virus. Their investigation revealed that the effect of miR-155 on the inhibition of DENV replication was due to target BTB domain and CNC homolog 1 (Bach1), resulting in the induction of the heme oxygenase-1 (HO-1)-mediated inhibition of DENV NS2B/NS3 protease activity and enhanced anti-viral IFN responses [43]. Interestingly, researchers have also suggested that the TLR4/NF-κB/miR-155-5p/SOCS-1 axis in human macrophages may be regulated by vitamin D during DENV infection [41].

West Nile virus (WNV) is a member of the *Flaviviviridae* family, with an ssRNA genome, which has disseminated globally and is a significant cause of viral encephalitis in humans [40,44]. It was reported that miR-155 was up-regulated in the brains of mice infected with WNV. This up-regulation was correlated with neuroinflammatory molecules and target genes for miR-155, including IL-13, brain-derived neurotrophic factor (BDNF), and C-C motif chemokine receptor 9 (CCR9) [45]. One of the target genes, IL-13, is involved in cell survival, and the reduced levels of IL-13 observed in WNV-infected mice may promote apoptosis [46]. Natekar et al. [47] demonstrated the critical role of miR-155 in WNV infection in mice. MiR-155 knockout (miR-155−/−) mice exhibited significantly higher morbidity and mortality after infection with a lethal strain. Increased mortality in miR-155−/− mice was associated with a significantly high WNV load in sera and brains. In the same mice, higher levels of protein IFN-α were observed, as well as significantly lower levels of anti-viral interleukins (i.e., IL-1β, IL-12, IL-6, and IL-15) and granulocyte-macrophage colony-stimulating factor (GM-CSF). Additionally, the over-expression of miR-155 in human neuronal cells modulated the anti-viral cytokine response, resulting in significantly lower WNV replication [47].

#### 2.1.3. Respiratory Viruses

Influenza virus (IV) belongs to the *Orthomyxoviridae* family, with an ssRNA genome [48]. Influenza is an acute infectious respiratory disease that occurs seasonally in temperate climates while, in tropical regions, it can occur year-round, thus causing epidemics. These viruses circulate in all parts of the world and cause influenza of varying severity, sometimes leading to hospitalization and even death [49]. Patients with severe influenza exhibit bilateral pulmonary infiltration and, often, acute lung injury (ALI) and acute respiratory distress syndrome (ARDS) with associated hypoxemic respiratory failure [50]. MiR-155 might serve as a positive pro-inflammatory regulator in the inflammatory response induced by influenza H1N1 infection in human pulmonary microvascular endothelial cells (HPMECs). In virus-infected HPMECs, the miR-155 level was significantly increased at 16 h and 24 h. Sphingosine-1-phosphate receptor 1 (S1PR1) can be targeted by miR-155, where the over-expression of miR-155 may decrease the expression of S1PR1, leading to NF-κB activation, cytokine production, and increased inflammatory response. An in vitro study showed that the over-expression of miR-155 in IV-infected pulmonary vascular endothelial cells promoted the expression of IFN-β, IL-1β, TNF-α, IL-6, IL-8, C-C motif chemokine ligand (CCL) 2, and CCL5; however, the down-regulation of miR-155 by an miR-155 inhibitor in infected cells promoted the expression of S1PR1, thus decreasing the levels of pro-inflammatory cytokines [51]. Another study indicated that the introduction of miR-155 into the non-structural gene segment of influenza A virus (IAV) increased the immunogenicity of the virus after experimental vaccination in mice. Immunization with the recombinant influenza virus promoted the proliferation of influenza-specific CD8 + T-cells and produced higher titers of neutralizing antibodies [52]. An in vivo study carried out in mice showed that miR-155 deficiency profoundly impairs in vivo CD8+ T-cell responses against viral infections. Additionally, miR-155 deficiency resulted in increased type I IFN signaling and reduced CD8+ T-cell proliferation, which may contribute to impaired viral clearance [53]. However, miR-155 may contribute to a more severe course of the influenza. Studies have demonstrated that miR-155 knockout mice recovered faster after influenza infection, with decreased lung inflammation, endoplasmic reticulum stress (ER stress), and pathological process, despite increased collagen expression [54].

Respiratory syncytial virus (RSV) belongs to the *Pneumoviridae* family, with an RNA genome [55]. RSV is the most important cause of viral lower respiratory tract illness (VLRTI) in infants, children, the elderly, and immunocompromised patients. Clinical manifestations of the disease range from asymptomatic infection to a form with bronchospasm and pneumonia [56]. Similarly to influenza virus infection, increased miR-155 expression has been observed in RSV infection [57,58,59]. The airway secretion of miR-155 during RSV infections in young children was associated with enhanced anti-viral immunity. These results provide evidence that miR-155 is strongly linked to high IFN-γ production and enhanced airway Th1 cytokine polarization (IFN-γ/IL-4 ratio). Additionally, high airway miR-155 levels have been linked to decreased respiratory disease severity [57]. Furthermore, increased miR-155 expression has been observed in the PBMCs of infants with RSV infection with pneumonia, which was positively related to the expression of TNF-α, IL-1β, IL-6, and IL-8 [59]. NF-κB activation following RSV-antigen binding to the pathogen recognition receptors TLR4 or retinoic acid-inducible gene 1 (RIG-1) is a primary stage in the immunological response to RSV, leading to increased miR-155 expression [58,60]. MiR-155 produced by targeting SHIP1 positively regulates myeloid proliferation, while that produced by the regulation of the p27^kip1^ protein level through the targeting of kinesin-like protein (Kif1) leads to dendritic cell maturation, which is a necessary step for dendritic cell migration to the lymph nodes and antigen presentation [61,62]. Interestingly, miR-155 may be a target for the treatment of RSV infection. Luteolin inhibited RSV replication in both in vitro and in vivo studies through the induction of miR-155, which targets SOCS1, leading to enhanced STAT1 phosphorylation and expression of interferon-stimulated genes (ISGs) [63].

Severe acute respiratory syndrome coronavirus 2 (SARS-CoV-2) belongs to the *Coronaviridae* family, with an RNA genome [64]. SARS-CoV-2 is the etiological agent of the coronavirus disease 2019 (COVID-19), an acute respiratory infectious disease. Most cases of COVID-19 have a benign course, but some can lead to life-threatening pneumonia and multiple organ failure [65]. In SARS-CoV-2 infection, hyper-inflammation, cytokine storm, and an inability to properly produce IFN are among the pathogenic mechanisms of coronavirus disease 2019 (COVID-19) [66,67,68]. In patients with COVID-19, an elevated expression of miR-155 has been observed in the nasopharynx, plasma, and PBMCs [69,70,71,72]. Interestingly, the miR-155 level has been found to be correlated with the severity of COVID-19, with the highest expression observed in non-surviving COVID-19 patients [69,71]. Furthermore, in SARS-CoV-2-infected cells, significant up-regulation of miR-155 has been observed in vitro [73,74]. Additionally, miR-155 has presented significant correlations with the clinicopathological characteristics of COVID-19 patients, such as chest computed tomography (CT) scan findings, C-reactive protein (CRP), ferritin, mortality, D-dimer, white blood cell (WBC) count, and lymphocyte and neutrophil percentages [69]. Donyavi et al. [71] demonstrated the existence of an inverse correlation between miR-155-5p and the SARS-CoV-2 N-gene and SARS-CoV-2 RdRp-gene, which may indicate that the expression of miR-155 increases due to an immune response to fight SARS-CoV-2. Based on bioinformatic analyses, it has been indicated that miR-155 may be associated with some genes involved in the immune response, apoptosis, and COVID-19 progression, such as STAT1, STAT3, transforming growth factor beta 1 (TGFB1), mothers against decapentaplegic homolog 3 (SMAD3), IRF1, AKT1, MYB proto-oncogene (MYB), BCL6, TP6, hypoxia inducible factor 1 subunit alpha (HIF1A), forkhead box P3 (FOXP3), AP-1 transcription factor subunit (JUNB), and nuclear factor kappa B subunit 1 (NFKB1) [71]. Qi et al. [75] suggested that human hsa-miR-155-5p might regulate the host immune response by controlling the expression of SOCS1, IL6, IL1B, colony stimulating factor 1 receptor (CSF1R), programmed death-ligand 1 CD274, TLR6, and TNF. Additionally, they hypothesized that the competing endogenous RNA (ceRNA) network could be utilized to modulate the host immune response to SARS-CoV-2 infection, such as the nuclear paraspeckle assembly transcript 1 (NEAT1)–miR-155-5p–IL-6/TNF/IL-1β axis; however, the specific mechanisms still need to be uncovered [75]. The in vivo suppression of miR-155 in SARS-CoV-2-infected mice transgenic for human angiotensin I-converting enzyme 2 receptor (tg-mice hACE2) improved survival and mitigated the cytokine storm and lung inflammation. This study reported that treatment with an miR-155 inhibitor improved the survival rate, promoted survival, and attenuated inflammation and the lung cytokine storm induced by the virus through the down-regulation of IL-1α, granulocyte colony-stimulating factor (G-CSF), IL-9, major intrinsic protein of lens fiber (MIP)-2, and interleukin 12 p70 (IL-12-P70), while increasing vascular endothelial growth factor (VEGF), interferon-induced protein 10 (IP-10), IFN-γ, monocyte chemoattractant protein-1 (MCP-1), C-X-C motif chemokine ligand 9 (MIG), MIP-1α, macrophage colony-stimulating factor (M−CSF), TNF-α, and MIP-1β production in lung secretions [72]. There have also been reports that miR-155, due to various correlations, could serve as a diagnostic clinical biomarker for the detection of COVID-19 and the severity of infection and, additionally, might be a predictor of chronic myocardial damage and inflammation in patients after COVID-19 [69,71,76].

Human adenovirus (HAdV) belongs to the *Adenoviridae* family, with a dsDNA genome [77,78]. It has a dramatic impact on its host cell, causing degenerative changes and necrosis of the epithelium of the respiratory tract, gastrointestinal tract, and conjunctiva. In severe cases of HAdV infection, systemic infection may occur [79]. Similarly to the infections described above, HAdV infection leads to the up-regulation of miR-155 [57,80]. Zhao et al. [80] showed that early stages of HAdV type 2 infection in human lung fibroblast cell culture led to increased miR-155 expression [80]. It has been speculated that miR-155 plays a role in the host anti-viral response, as an early gene in primary macrophages responding to different types of inflammation mediators (e.g., cytokine IFN-β) [80,81]. Additionally, miR-155 may inhibit SOCS1, subsequently enhancing type I IFN effector gene expression and the type I IFN-mediated anti-viral response [82]. 

Human rhinovirus (HRV) belongs to the *Picornaviridae* family, with an ssRNA genome [83]. HRV is the etiological agent of most common colds in healthy subjects, and is a major trigger of chronic obstructive pulmonary disease (COPD) and asthmatic exacerbations, representing a significant problem for disease management [84]. In young children, HRV infection has been linked with the airway secretion of extracellular vesicles containing miR-155, promoting local Th1 cell-mediated anti-viral responses. MiR-155 targets a set of target genes (similar to those in HAdV infection) which control the immune responses to rhinoviruses [85]. Additionally, miR-155 may act as a negative regulator of SHIP1, hence enhancing IFN type I signaling [20]. Bondanese et al. [84] have suggested that the over-expression of miR-155 in human bronchial epithelial cells can affect human rhinovirus 1B (HRV1B) replication by inhibiting its replication, thus participating in the anti-viral response.

In conclusion, miR-155, as the main immune regulator, plays an extremely important role in a range of viral infections. However, the most important role of miR-155 is in the regulation of genes related to the immune response, including SOCS1, SHIP1, and TNF, which influence the regulation of the production of IFN types I and II. Additionally, researchers have noted the ability of miR-155 to inhibit the replication of viruses through the regulation of genes related to the anti-viral response and oxidative stress.

### 2.2. MicroRNA-223 (miR-223)

MiR-223 plays a key role in the development and homeostasis of the immune system. To date, miR-223 has been demonstrated to be involved in many types of cancers, inflammatory diseases, autoimmune diseases, and other pathological processes [86]. Studies have shown that miR-223 expression is mainly altered during the inflammatory response of various cell types, including granulocytes, macrophages, dendritic cells (DCs), T-cells, endothelial cells, and epithelial cells. Alterations in miR-223 expression regulate the various functions of these cells, attenuating or exacerbating the associated tissue inflammation, thus also playing a role during viral infection [87,88].

#### 2.2.1. Hepatitis Viruses

MiR-223 is abundantly expressed in the liver, and may play a role in the pathology of viral hepatitis. A study revealed elevated serum miR-223 not only in patients with HCC, but also in patients with chronic hepatitis B (CHB) [89]. Meanwhile, in liver tissue with HCC, miR-223 has been found to be down-regulated [90]. This may be due to the fact that hepatocytes contain abundant miR-223, and their damage—for example, caused by inflammation due to viral infection or cancer—can be expected to lead to the release of a significant amount of this miRNA into the circulation [89]. However, another study has shown lower miR-223 levels in the sera of HBV-positive patients than in healthy controls [91,92]. In vitro studies have demonstrated that miR-223 is down-regulated in HBx- or HBV-transfected HepG2 cells, as well as in HepG2.2.15 cells, and the repression of miR-223 has been associated with the up-regulation of c-Myc in infected cells [92]. 

Furthermore, during HCV infection, the level of miR-223 in serum was decreased [93]. In addition, liver biopsies from CHC patients presented lower miR-223 levels. Notably, low miR-223 levels likely contribute to chronic liver inflammation and subsequent complications through targeting of the NF-κB pathway during viral infection [94]. It has been potentially indicated that this miRNA has an anti-viral function due to high levels of miR-223 being detected in patients who had reached a sustained virological response after treatment [93]. Thus, researchers have suggested that miR-223 might serve as a novel and potential non-invasive biomarker correlated with the therapeutic effect and pathological features of liver disease [92,93].

#### 2.2.2. Hemorrhagic Viruses

Similarly to the viruses discussed earlier, DENV has also been shown to decrease the miR-223 levels in infected cells. Moreover, a study showed that miR-223 over-expression in a human endothelial-like cell line inhibited DENV2 replication. Further analysis showed that miR-223 directly targeted the 3′UTR of the mRNA for microtubule-destabilizing protein stathmin 1 (STMN1), thereby reducing its mRNA and protein levels [95]. The study also indicated that STMN1 is up-regulated during DENV2 infection and, so, the inhibition of STMN1 expression had a negative effect on DENV replication. Moreover, researchers showed that decreased C/EBPα and increased E2F transcription factor 1 (E2F1) played a major role in the DENV2-induced down-regulation of miR-223 through a negative feedback loop. The results suggested that miR-223 downregulation may be associated with the induction of pro-inflammatory stimuli, and that miR-223 itself may act as a novel anti-viral factor, which may open an avenue to limiting DENV infection [95].

#### 2.2.3. Respiratory Viruses

Studies have indicated that miR-223 is differentially expressed in response to influenza viruses, and may contribute to the virulence of the influenza A virus (IAV) [96,97,98]; in particular, during IAV infection, the up-regulation of miR-223 in lung tissue, whole blood, and infected cells has been observed [96,97,99]. In addition, a higher level of miR-223 was observed with infections causing severe and lethal pulmonary disease, such as those of r1918 (the reconstructed 1918 influenza virus), A/Vietnam/1203/04, or H5N2 ma81, in contrast with non-lethal infections of some other H1N1 influenza virus strains, including A/Texas/36/91 (Tx/91), A/Kawasaki/173/01 (K173), or H5N1 w81 [96,97,100]. A study showed that, along the duration of the infection, the up-regulation of miR-223 occurred [100]. The up-regulation of miR-223 was also observed in 2009 pandemic H1N1 virus and PR8 infection [98]. Interestingly, research showed that the inhibition of miR-223-3p reduced IV replication in the lungs, while over-expression of this miRNA in the lungs augmented the infection. Mice treated with anti-miR-223-3p presented increased survival and started to gain weight 6 days after infection [100]. Moreover, studies have indicated that increased miR-223 is associated with the regulation of inflammation in mouse lungs through down-regulation of the pro-inflammatory cytokine production of TNF-α [97,101], and also promotes granulocytic differentiation [100]. Additionally, the upregulation of miR-223 may repress the activity of cyclic AMP responsive element-binding protein (CREB)—which is a transcription factor involved in critical functions, including T-cell development and cell survival—by targeting PI3K, insulin-like growth factor 1 receptor (IGF1R), G protein coupled receptors (GPCR), protein phosphatase 2 (PP2A), cAMP-dependent protein kinase (PKA), and the Ca^2+^ channel [96]. Other analyses have shown that miR-223 may regulate interleukin 1 receptor antagonist (IL1RN), melanoma differentiation-associated protein 5 (MDA5), and STAT1 genes, and can participate in the regulation of cell death and apoptosis [100]. The research results obtained by Liu et al. [102] indicated that miR-223 was down-regulated in patients during IAV infection and in cell cultures infected with H1N1 (A/Puerto Rico/8/34). Further research showed that miR-223 over-expression by a mimic had inhibitory effects on IAV-induced elevated ROS intensity, inflammatory cytokine (TNF-α, IL-1β, IL-6, and IL-18) contents, and cell apoptotic rate, and also lowered the expression of NOD-like (NLR) receptor family pyrin domain containing 3 (NLRP3) [102], the over-expression of which can lead to a disordered inflammatory state and the immune pathogenesis of cells under stimulus [103]. 

Severe acute respiratory syndrome coronavirus (SARS-CoV) is a member of the *Coronaviridae* family, with an RNA genome [64]. SARS-CoV is the etiological agent of severe acute respiratory syndrome (SARS). It is characterized by severe symptoms associated with lower respiratory tract infection, causing alveolar damage. Atypical pneumonia with rapid deterioration and failure may occur due to increased levels of activated pro-inflammatory chemokines and cytokines. In severe cases of SARS, ARDS may be observed as a severe life-threatening immune-mediated disease [104]. In the case of SARS-CoV infection, miR-223 is down-regulated in bronchoalveolar stem cells (BASCs), which may contribute to the development of infection. Mallick et al. [105] indicated that the N and S proteins of SARS-CoV down-regulate miR-223, which may help the N protein to enter the host cell. Furthermore, decreased expression of miR-223 may help SARS-CoV to escape miRNA-mediated repression, rescuing the virus for effective transmission at the initial stage of viral infection. In addition, researchers have suggested that, upon successful entry, the N protein may use miR-223 once again to activate C-C motif chemokine receptor 1 (CCR1), the inflammatory chemokine receptor for CCL3 and CCL5, via NF-κB at its replicative stage, in order to enhance lung fibrosis [105].

As with SARS, miR-223 is decreased in SARS-CoV-2 infection [106,107]. Additionally, it has been predicted that miR-223 directly binds to the receptor of the S protein, mediating the membrane fusion and entry of SARS-CoV-2 into cells. An in vitro study indicated that an miR-223-3p mimic significantly inhibited the expression of the S protein, and also significantly inhibited SARS-CoV-2 replication [106]. Due to the fact that important targets for miR-223 involved in infection and inflammation are TNF receptor-associated factor 6 (TRAF6), forkhead box O1 (FOXO1), TLR4, STMN1, PI3K/AKT, C-X-C motif chemokine ligand 2 (CXCL2), CCL3, IL-6, IFN-I, IL-1β, Caspase-1 (and, mainly, NLRP3), inhibitor of nuclear factor kappa-B kinase subunit alpha (IKKα), and NF-κB, it participates in regulating the inflammatory process while also indicating antioxidant and anti-viral roles, suggesting miR-223 as a potential regulatory factor in the process of COVID-19 immunopathogenesis [108].

#### 2.2.4. Human Immunodeficiency Viruses

Human immunodeficiency virus-1 (HIV-1) is a member of the *Retroviridae* family, with an ssRNA genome [109]. Human immunodeficiency virus (HIV) causes acquired immune deficiency syndrome (AIDS), which kills or impairs cells of the immune system and progressively destroys the body’s ability to fight infections and certain cancers. RNA viruses, such as HIV-1, are replicated in the cytoplasm. Given that miRNAs primarily regulate mRNA in the cytoplasm, it is possible that miRNAs function as anti-viral factors by directly targeting and controlling the replication of RNA viruses. Huang et al. [110] showed that miR-223 potently inhibits HIV-1 production in resting primary CD4+ T-cells [110]. The reduction of miR-223 or inhibition of its activity accelerated HIV-1 replication in dormant CD4+ T-cells [110,111]. A higher miR-223 level was also observed in monocytes, whereas HIV-1 infectivity was greater in macrophages than in monocytes, potentially indicating a role of miR-223 in regulating the susceptibility of immune cells [111]. However, Sun et al. [112] pointed out that, due to the low expression levels of miR-223 in T-cells and the poor accessibility of the target site, it is unlikely that miR-223 directly inhibits HIV-1 in these cells. Meanwhile, the expression of miR-223 is significantly increased in myeloid, cells which may indicate that it has an inhibitory function on HIV-1 in NK cells, macrophages, and monocytes. It is more likely, though, that miR-223 influences HIV-1 infection by targeting cellular factors required for replication, such as ras homolog family member B (RhoB), Sp3 transcription factor (Sp3), and leukemia inhibitory factor (LIF) [112]. RhoB functions as an anti-apoptosis gene, and can activate the AKT–NF-κB pathway. It has also been reported that RhoB is down-regulated in HIV-1-infected cells obtained from patients. Sp3 can repress HIV-1 LTR activity directly, and may also activate apolipoprotein B mRNA editing enzyme catalytic subunit 3G (APOBEC3G), which is a host viral restriction factor. LIF can restrict HIV-1 replication, and has been reported to be down-regulated in early HIV-1 infection. Therefore, miR-223 may function as a negative factor in HIV-1 infection by reducing RhoB-mediated activation of the AKT–NF-κB pathway. This miRNA may also function as a positive factor in HIV-1 infection by targeting HIV-1-suppressive Sp3 and LIF [112].

In conclusion, miR-223 plays an important role in the regulation of the immune response. Altered expression of miR-223 is commonly observed during viral infections, which is crucial for maintaining immune homeostasis to avoid excessive inflammation and tissue damage as a result of the infection. In addition, miR-223 over-expression inhibits replication of viruses, which may constitute a good basis for the development of new anti-viral therapies in the future.

### 2.3. MicroRNA-146a (miR-146a)

MiR-146a, as a modulator of the differentiation and function of cells of innate as well as adaptive immunity, has particular importance. In T-cells, miR-146a might be involved in the determination of the fate of Th1 and Th2 cells due to their differential expression [113]. In addition, miR-146a is induced by T-cell receptor (TCR) stimulation in memory T-cells and has also been shown to be critical for Treg functions [114,115]. Furthermore, miR-146a is quickly induced upon the activation of human monocytes, and is also inducible by inflammation in an NF-κB-dependent manner, targeting the TRAF6 and IL-1 receptor-associated kinase (IRAK) 1 genes [114]. Studies have reported that negative regulation of miR-146a may contribute to promoting viral replication in macrophages through type I IFN down-regulation in an RIG-I/NF-κB-dependent manner, and the down-regulation of TRAF6, IRAK1, and IRAK2 [116]. Considering the above, miR-146a seems to be an important regulator in viral infection. MiR-146a plays a significant role in inflammatory settings and in the T-lymphocyte-mediated adaptive immune response, which has a pivotal role in viral infections [117,118].

#### 2.3.1. Hepatitis Viruses

In CHB and acute chronic liver failure (ACLF) caused by HBV, miR-146a-5p expression levels have been observed to be up-regulated in sera and in a human hepatic cell line [118,119,120]. HBV infection led to higher levels of miR-146a and pro-inflammatory cytokines (e.g., TNF-α, IL-6, IL-8, IL-12, and IL-18), along with a reduction in X-linked inhibitor of apoptosis (XIAP) expression via NF-κB activation [118]. In chronic HBV infection, inflammatory cytokines and viral factors may induce miR-146a expression in T-cells. The up-regulation of miR-146a may subsequently lead to the suppression of the anti-viral function of CD4+ and CD8+ T-cells by targeting STAT1, and may contribute to the persistence of the virus. MiR-146a also decreases the cytotoxicity of T-cells, as well as the production of anti-viral cytokines (e.g., IFN-γ, IL-2, and TNF-α). Additionally, an in vitro study has shown that the blockage of miR-146a in T-cells in CHB greatly enhanced virus-specific T-cell activity [117]. Li et al. [121] found that HBx promoted the expression of miR-146a through the NF-κB signaling pathway, and that increasingly expressed miR-146a down-regulated its target, complement factor H (CFH)—an important negative regulator of the alternative complement pathway. These results demonstrate that the HBx–miR-146a–CFH–complement activation regulation pathway might play an important role in the immunopathogenesis of chronic HBV infection and the promotion of liver inflammation [121]. Interestingly, a single-nucleotide polymorphism within the miR-146a gene may contribute to acute-on-chronic hepatitis B liver failure. Jiang et al. [122] reported that the GG genotype within the pre-miR-146a in PBMCs was associated with higher expression of miR-146a, lower levels of serum TNF-α concentration, and a relatively higher survival rate [122]. Research has shown that miR-146a may affect HBV replication [118,119,120]. It has been reported that HBV induces the autophagic response of the miR-146a-5p–XIAP–mouse double minute 2 homolog (MDM2)/cellular tumor antigen p53 (p53) pathway, leading to enhanced HBV replication [118]. Another target of miR-146a affecting HBV replication is zinc finger E-box-binding homeobox 2 (ZEB2) [119], which has been found to suppress HBV transcription and replication by targeting its core promoter [123]. The in vitro study of Wang and Li [119] demonstrated that the over-expression of miR-146a or knockdown of ZEB2 promoted HBV replication and expression, while the down-regulation of miR-146a or over-expression of ZEB2 suppressed viral replication [119]. MiR-146a may also regulate HBV replication indirectly through flap endonuclease 1 (FEN1), which repairs relaxed circular DNA to form covalently closed circular DNA, thus promoting HBV DNA replication [120,124]. The regulation of FEN1 takes place through the down-regulation of TRAF6 and IRAK1 by miR-146a, decreasing the activity of the NF-κB pathway. In addition, Argonaute-2 (Ago2) cooperates with miR-146a to regulate the transcription and expression levels of the FEN1 protein through the downstream target gene IRAK1/TRAF6, promoting HBV replication [120]. 

MiR-146a has also been found to be up-regulated in monocytes from HCV-infected patients [125,126]. Zhang et al. [126] indicated that the HCV core protein could promote the expression of miR-146a through the TLR2–myeloid differentiation primary response 88 (MyD88) pathway [126]. However, the level of miR-146a in monocytes with HCV infection decreased following TLR stimulation [125]. Another mechanism regulating miR-146a expression in hepatocytes during HCV infection is NF-κB signaling [127]. Another study showed that the inhibition of miR-146a in monocytes from HCV-infected patients led to decreased IL-23, IL-10, and TGF-β expression through the induction of the SOCS1/STAT3 pathway [125]. Ren et al. [125] have suggested that miR-146a increases cytokine production in monocytes and increases regulatory T-cells during HCV infection through SOCS1/STAT3 signaling induction, which may lead to immune injury of the liver during chronic viral infection [125]. As in HBV infection, miR-146a-5p promotes HCV infection by enhancing the production of infectious viral particles and, furthermore, contributes to the development of liver disease and HCC by targeting genes associated with inflammation, fibrosis, and cancer development, including CXC motif chemokine receptor 4 (CXCR4), TLR2, TRAF6, IRAK1, breast cancer gene 1 (BRCA1), NFKB1, epidermal growth factor receptor (EGFR), CD40 ligand (CD40LG), SMAD4, hepatocyte nuclear factor 1 homeobox A (HNF1), SHIP1, and TLR4 [127].

#### 2.3.2. Hemorrhagic Viruses

DENV infection induces massive immune activation and the production of high amounts of pro-inflammatory cytokines, which can be regulated by miR-146a [128]. Wu et al. [129] found that expression of miR-146a was significantly up-regulated after DENV-2 infection of human primary monocytes and THP-1 cells. Moreover, they indicated that the over-expression of miR-146a inhibited the production of IFN-β and IL-28A/B by TRAF6, and also contributed to increased DENV2 replication [129]. MiR-146a, by targeting TRAF6, also decreased the conversion of the microtubule-associated protein light-chain 3-I (LC3-I) to LC3-II, as well as negatively regulating the autophagy process of A549 cells and THP-1 cells during DENV2 infection, thus contributing to efficient viral replication [130]. Similarly, up-regulation of miR-146a has been observed in the hepatic tissue of patients with dengue hemorrhagic fever, which may promote the inflammatory response and pathological liver damage [131]. Interestingly, Ouyang et al. [132] demonstrated that miR-146a was significantly decreased in the sera of dengue patients. Additionally, they showed that miR-146a-5p was negatively correlated with serum aspartate aminotransferase (AST) and ALT activities in dengue-infected patients, suggesting that miR-146a might mediate the development of liver inflammation. However, the physiological or pathological significance of reduced miR-146a in the sera of dengue-infected patients remains ambiguous and, thus, requires further research [132].

#### 2.3.3. Respiratory Viruses

Research has indicated that miR-146a is induced during IAV infection and, so, may play an important role in viral replication in this context [133,134,135,136]. Zhang et al. [133] showed that the over-expression of miR-146a in A549 cells diminished IFN type I (IFN-I) responses by decreasing IFN-β production and ISG expression, thereby promoting IAV replication [133]. In addition, they found that miR-146a directly targets TRAF6, which is involved in the production of IFN-I, where TRAF6 over-expression reversed the replication-promoting effect of miR-146a on IAV. Furthermore, the in vivo inhibition of miR-146a alleviated IAV-induced lung injury in mice and enhanced survival rates by promoting type I IFN anti-viral activities [133]. Another study has indicated that, during IAV infection, miR-146a may negatively regulate IRAK1 and exert an influence on the neurotrophin and Toll-like signaling pathway, IL-7 signaling pathway, VEGF signaling pathway, or JAK-STAT pathway [134]. Functional analysis has additionally revealed that miR-146a is strongly associated with the innate immune response, cytokine production, and apoptosis [135]. In human nasal epithelial cells (hNECs), after infection with influenza H3N2 virus, miR-146a was induced, regulating TRAF6 expression but not IRAK expression in the nasal epithelium; in contrast, it targeted IRAK1 in the lower airway [136]. Interestingly, Terrier et al. [135] indicated that the inhibition of miR-146a significantly increased viral propagation. Therefore, more research is needed to clarify the anti-viral effect of miR-146a in IAV infection and influenza–host interactions. 

As in the case of IAV infection, RSV infection strongly increased the expression of miR-146a-5p [137]; however, in vitro studies have shown that miR-146a targets 70 kilodalton heat shock proteins (HSP-70) during RSV infection, and not (as with IAV) TRAF6 and IRAK1, which may have an effect on viral replication [137,138]. 

In contrast to other respiratory infections, during SARS-CoV-2 infection, miR-146a down-regulation has been observed, where the level of miR-146a was correlated with the severity of COVID-19 [139,140]. Tang et al. [139] have pointed out that miR-146a plays fundamental roles during SARS-CoV-2 infection by targeting TRAF6, IRAK1, and IRAK2 which participate in the NF-κB pro-inflammatory signaling pathway in immune cells. Additionally, analysis has revealed the broad up-regulation of STAT1, targeted by miR-146a-5p, which encodes a key element of the JAK/STAT pathway [139]. The miR-146a deficiency observed in diabetes leads to enhanced inflammation and increased synthesis of MCP-1, followed by further reduction in miR-146a by enhancing the signaling of systemic effects by TGF-β1/Erb-B2 receptor tyrosine kinase 4 (ErbB4)/neurogenic locus notch homolog protein 1 (Notch1) accompanying severe COVID-19; this may explain the more severe COVID-19 cases occurring in these patients [141]. However, in the oral fluids of patients with type 2 diabetes, increased levels of miR-146a have been observed, which may lead to the up-regulation of angiotensin converting enzyme 2 (ACE2) expression; these are essential SARS-CoV-2 receptors, and modulate the host anti-viral response in these patients [142]. Sabbatinelli et al. [140] showed that COVID-19 patients presented increased IL-6 levels and reduced miR-146a-5p levels compared to healthy age-matched subjects, pointing out the imbalance in the IL-6/miR-146a-5p physiological axis in the pathogenesis of SARS-CoV-2 infection [140]. Additionally, patients with multi-focal interstitial pneumonia due to SARS-CoV-2 infection who did not respond to anti-IL-6 therapy had lower serum levels of miR-146a-5p, and experienced a more severe course of the disease [140]. The down-regulation of miR-146a in COVID-19 may cause hyperactivation of the immune response, a loss in T-cell function, and immune dysregulation in patients with severe COVID-19 and, so, it might be key regulator of COVID-19 pathogenesis [139]. Moreover, the down-regulation of miR-146a may cause excessive cytokine production and the lack of a feed-back mechanism to limit inflammatory damage to tissues [141].

In conclusion, miR-146a regulates genes influencing the activity of the NF-κB transcription factor and, thus, may affect the production of pro-inflammatory cytokines during viral infections. Additionally, miR-146a indirectly influences viral replication by regulating NF-κB activity.

### 2.4. MicroRNA-122 (miR-122)

MiR-122 is an miRNA that is conserved among vertebrate species. MiR-122 typically has a liver-enriched expression and is one of the most abundant miRNAs in the liver, accounting for about 70% and 52% of the whole hepatic miRNome in adult mice and humans, respectively [143,144]. MiR-122 is a central player in liver biology (including liver development) and differentiation, supports spontaneous regeneration, and takes part in liver homeostasis, lipid metabolism, and cholesterol synthesis [145,146,147]. Hence, alterations in intrahepatic miR-122 have been associated with liver disease, including hepatitis with viral etiology (HBV, HCV), steatosis, cirrhosis, and hepatocellular carcinoma (HCC) [147]. 

#### 2.4.1. Hepatitis Viruses

As liver-specific miRNA, miR-122 has been found to be closely related to HBV replication and liver injury [148]. MiR-122 expression in the liver was significantly down-regulated in patients with HBV infection compared with healthy controls, and the miR-122 levels were negatively correlated with intrahepatic viral load and hepatic necroinflammation [149]. Additionally, Wu et al. [150] showed that serum miR-122 levels were significantly lower in patients who developed a virological response (VR) compared with the non-VR group [150]. The decreased miR-122 expression in HBV can lead to increased expression of the cyclin G1 gene. Then, cyclin G1 can attenuate the activity of P53, which increases HBV replication; therefore, a loss in miR-122 expression in HBV patients may activate modulating cyclin G1 and increase HBV replication [149]. On the other hand, it has been reported that serum miR-122 levels were positively associated with serum HBV DNA levels in chronic hepatitis B (CHB) patients [150]. Ebrahimifard et al. [151] also showed that miR-122 levels in sera from CHB patients were higher than those of the control group [151]. Van de Ree et al. [152] shown that plasma miR-122 levels were approximately 60 times higher in CHB patients compared to healthy controls, and approximately 2 times higher in hepatitis B antigen (HBeAg)-positive patients vs. HBeAg-negative patients [152]. Such increases in circulating miR-122 levels in plasma might be caused by the HBV-induced up-regulation of miR-122 expression and increased secretion from the liver [152]. In an in vitro study, researchers reported that the transfection of an miR-122 mimic inhibited HBV expression, whereas the anti-sense inhibition of miR-122 led to increased HBV production in transfected cells. In another study, it was observed that the down-regulation of HO-1 by miR-122 played a negative role in the miR-122-mediated inhibition of viral expression [153,154,155]. MiR-122 can also inhibit HBV replication by modulating the expression of type I IFN, which can play a significant role in the host anti-viral response, such as protecting against HBV infection [156]. The activity of the JAK/STAT signaling pathway can be negatively regulated by SOCS, and miR-122 may also down-regulate SOCS3 [157]. Although the expression of miR-122 is transcriptionally regulated by liver-enriched transcription factors, including hepatocyte nuclear factor 4 alpha (HNF4) and C/EBPα, it may also be regulated by viral infection [145,158,159]. The transfection of HBV genes into liver cells (Huh7 and HepG2) suppressed the expression of miR-122, which subsequently induced the expression of apolipoprotein B mRNA-editing enzyme catalytic subunit 2 (APOBEC2). These findings further suggest that the effects of HBV on APOBEC2 occur via the down-regulation of cellular miR-122 expression, which may contribute to the tumorigenesis of liver cells [160]. Additionally, research has suggested that hepatitis B virus X protein (HBx) is an important negative regulator of miR-122 expression, highlighted by the fact that HBx binds to peroxisome proliferator activated receptor gamma and inhibits the transcription of miR-122 [92,161]. Another study has shown that the down-regulation of miR-122 may involve HBx through the down-regulation of germline development 2 (Gld2) [162]. More interestingly, other studies have found that serum miR-122 levels may serve as an indicator for viral translation and a potential marker for risk stratification in patients infected with HBV [163,164].

MiR-122 is an abundant, liver-specific miRNA that is an unusual host factor for HCV, an important cause of liver disease in humans [165]. MiR-122 has been shown to be required for the replication of HCV in the hepatoma cell line Huh7. Jopling et al. [166] have shown that sequestration of miR-122 leads to a marked loss in replicating viral RNAs, and simultaneous recognition of the binding site within the 5′-NCR by miR-122 is required for miR-122-induced viral RNA accumulation; this suggests that miR-122 is likely to facilitate viral RNA replication through interaction with the viral 5′-NCR [166]. There have been reports that miR-122 could also enhance HCV replication in non-hepatic human embryonic kidney epithelial cells (HEK-293) [167]. Interestingly, miR-122 expression has been shown to endow the ability of supporting efficient HCV RNA replication, virion production, and virion release in HepG2 cells. These results support the notion that miR-122 is required for HCV RNA replication, but does not greatly enhance viral translation [168]. Henke et al. [169] have shown that miR-122 stimulates HCV RNA translation at an early stage of association with the small ribosomal sub-unit in the viral RNA. The sequestration of miR-122 in liver cell lines strongly reduced HCV translation, whereas the addition of miR-122 stimulated HCV translation in liver cell lines, as well as in non-liver HeLa cells and in rabbit reticulocyte lysate. This stimulation effect was confirmed by the direct interaction of miR-122 with two target sites in the 5′-UTR of the HCV genome [169]. It has also been shown that miR-122 binds to HCV RNA in association with the Ago2 protein complex, slowing the decay of the viral genome in infected cells. Data have demonstrated that an RISC-like complex mediates the stability of HCV RNA, suggesting that Ago2 and miR-122 act in a coordinated manner to protect the viral genome from the 5′ exonuclease activity of the host mRNA decay machinery. Thus, miR-122 acts in an unconventional way to stabilize HCV RNA and slow its decay, expanding the repertoire of mechanisms by which miRNAs modulate gene expression [165]. In another study, the results of an Huh7 cell experiment indicated that the silencing of miR-122 with antagomir decreases HCV RNA abundance, whereas the transfection of miR-122 mimics increases the HCV level. Additionally, the antagomir of miR-122 up-regulates heme oxygenase-1 (HO-1), likely by decreasing transcription repressor Bach1, where HO-1 significantly inhibits HCV replication [170]. In addition, miR-122 has also been shown to be regulated by IFN; therefore, the down-regulation of its expression by IFN could serve as a general anti-viral mechanism. However, due to the cell-type-specific expression of miR-122, this effect may be limited to viruses that infect hepatocytes, such as HCV [171]. Researchers have also shown that the HCV core expression activates an miR122–transforming growth factor β receptor-associated protein 1 (TGFBRAP1) signaling pathway, which might be associated with promoting HCC progression [172]. Additionally, attention should be paid to circulating miR-122 as a diagnostic marker for chronic viral hepatitis detection [164]. In patients with CHC, the serum level of miR-122 has been found to be strongly correlated with serum ALT activity, as well as with necroinflammatory activity in patients with CHC and elevated ALT levels; however, this was not correlated with the fibrosis stage or functional capacity of the liver [173]. However, in the future, well-designed, large-scale, and accurate research is still needed to expand knowledge in this context.

#### 2.4.2. Hemorrhagic Viruses

The liver is one of the most important target tissues in severe cases of dengue due to its intense viral replication and metabolic role. Determining the roles of miRNA during infection is crucial in order to understand the regulatory mechanisms of DENV infection. De Oliveira et al. [131] studied the expression profile of miR-122 in liver tissue under dengue hemorrhagic fever (DHF). They observed a down-regulation of miR-122 in fatal cases compared to controls. Additionally, they selected target genes for miR-122 in the course of dengue, such as cytochrome P450 family 7 subfamily A member 1 (CYP7A1), IGF1R, serum response factor (SRF), Rac family small GTPase 1 (RAC1), Ras homolog family member A (RHOA), and cyclin G1 (CCNG), which are indirectly involved in immune processes [131]. Meanwhile, another study revealed the up-regulation of miR-122 in sera. The highest level of miR-122 was observed in patients with DHF. Elevated serum levels of miR-122 may be due to its release from damaged hepatocytes as a result of dengue infection [174]. MiR-122 may also modulate the replication of the DENV replicon. In hepatic Huh-7 cells, endogenous miR-122 effectively suppressed the translation levels of replicon D2R2A-30 X/122pmT before entering a more vigorous replication process, whereas stable expression of miR-122 in baby hamster kidney fibroblast (BHK)-21 cells effectively reduced the viral replication of D2R2A-30X/122pmT [175]. 

In the early stage of Ebola virus (EBOV) infection of Huh-7 cells with the RESTV strain, the expression level of miR-122 was reduced by half. Meanwhile, at 96 h of infection, a two-fold increase in miR-122 was observed in cells infected with the ZEBOV strain [176]. These studies also demonstrated that miR-122 can target the viral genomes of ZEBOV and RESTV; namely, the viral structural protein vp40 gene, which is responsible for regulating viral transcription and coordinating virion assembly [176,177].

#### 2.4.3. Respiratory Viruses

MiR-122 may be also regulated in respiratory viral infections. In the peripheral blood of infants infected with RSV, the down-regulation of miR-122 was observed. Pathway analysis indicated that the dysregulated miRNA was involved in inflammatory and immune responses, including Wnt [178]. Additionally, as a target gene of miR-122-5p, interleukin-1 receptor type I (IL1R1) may be activated by increased interleukin-1 receptor antagonist (IL1RA) after RSV infection, which is one of the RSV-induced genes [179,180]. Another target gene of miR-122-5p is TLR4, which is stimulated by the RSV F protein [180]. TLR4-deficient mice infected with RSV exhibited enhanced disease, and the expression of IL-13, TGF-β, and IL-6 was induced [181,182]. In addition to pulmonary symptoms, RSV can also present with cardiovascular symptoms [183]. Infants with severe acute bronchiolitis-caused RSV and coexisting cardiac dysfunction presented a sharp elevation in serum miR-122. In the case of RSV with cardiovascular disorders, the up-regulation of miR-122 may down-regulate the expression of cationic amino acid transporter-1 (CAT-1) or/and prevent the translation of inducible nitric oxide synthase (iNOS) mRNA, thereby weakening the anti-viral effect [184]. 

Collison et al. [185] found that HRV infection induced the expression of miR-122 in mouse lungs and human airway epithelial cells. The in vivo inhibition of miR-122 in the lung reduced neutrophilic inflammation and CXCL2 expression, enhanced the innate IFN response, and ameliorated airway hyper-reactivity in the absence and presence of allergic lung inflammation. Additionally, the inhibition of miR-122 in the lung increased the level of suppressor SOCS1, which is an in vitro-validated target of miR-122. Importantly, the gene silencing of SOCS1 in vivo completely reversed the protective effects of miR-122 inhibition on HRV-induced lung disease. These results suggest that miR-122 promotes HRV-induced lung disease through the suppression of the target gene SOCS1 in vivo [185].

In summary, miR-122—considered a liver-specific miRNA—plays a major role in the replication of hepatitis and EBOV viruses. During infection with hepatitis viruses, miR-122 can serve as a biomarker of viral infection. On the other hand, the data highlighted in our review indicate that the role of miR-122 in other viral infections related to the regulation of genes involved in innate immune response is poorly understood at present.

### 2.5. MicroRNA-125b (miR-125b)

MiR-125b is involved in regulating NF-κB, p53, PI3K/Akt/mTOR, v-erb-b2 avian erythroblastic leukemia viral oncogene homolog 2 (ErbB2), Wnt, and other signaling pathways, thereby controlling cell proliferation, differentiation, metabolism, and apoptosis [186]. However, the expression of miR-125b-5p may be also modulated by NF-κB signaling. MiR-125b negatively regulates the inflammatory response by targeting TNF-α [187]. In addition, inducing the inflammation of human macrophages in vitro through lipopolysaccharides (LPS) can suppress the expression of miR-125b [188]. It has been shown that miR-125b may inhibit the expression of a gene encoding 5-lipoxygenase—a key enzyme in the biosynthesis of leukotrienes, which are essential for the innate immune response and inflammatory processes [189]. Researchers have also demonstrated that miR-125b can promote macrophage-mediated inflammation. MiR-125b, by targeting interferon regulatory factor 4 (IRF4), potentiates the functional role of macrophages in inducing immune responses and anti-tumor activities [190]. Other direct target genes of miR-125b are p53 and p38 mitogen-activated protein kinase 1 (MAPK), which can activate the apoptotic pathway and induce mitochondrial apoptosis, respectively [186,191]. Furthermore, the levels of induced myeloid leukemia cell differentiation protein (Mcl-1), B-cell lymphoma-extra-large (Bcl-xL), and Bcl-2-like protein 2 (Bcl-w)—the anti-apoptotic members of the B-cell lymphoma 2 (Bcl-2) family—are increased by miR-125b [192,193]. MiR-125b also regulates T-cell proliferation and activation, is highly expressed in naive CD4 T-cells, and can inhibit the T-cell immune response; however, it may also promote T-cell apoptosis [186].

#### 2.5.1. Hepatitis Viruses

MiR-125b plays an important role in HBV and HCV infection, as well as in HCC development. Research has indicated that miR-125b-5p over-expression increased HBV replication in HepG2.2.15 cells [194,195]. However, Deng et al. [194] found that miR-125b increased HBV replication without altering HBV transcription. Their data demonstrated that miR-125b-5p targeted the LIN28B/let-7 axis in order to stimulate HBV replication in a post-transcriptional step [194]. In addition, miR-125b in serum has been positively correlated with the serum HBV DNA level and with grades of liver necroinflammation [195]. Meanwhile, plasma levels of miR-125b were remarkably decreased in HBV-HCC patients compared to healthy controls and HBV subjects without HCC. Moreover, the low plasma miR-125b levels in HBV-HCC patients were associated with a higher prevalence of metastasis [196]. In addition, it has been reported that miR-125b-5p inhibits the phosphorylation of retinoblastoma protein and blocks cell cycle progression at the G1/S phase in hepatoma cell lines [194]. An in vitro study by Zhang et al. [197] has shown that miR-125b regulates HBV expression by targeting the sodium channel, non-voltage-gated 1 alpha (SCNN1A) gene, and inhibits HBV core protein expression, as well as HBsAg and HBeAg secretion. Additionally, it has been shown that HBV infection of Hep.G2 cells reduces the level of miR-125b [197]. Due to the importance of the data presented, further research is needed to elucidate the role of miR-125b in HBV infection and its potential role as an anti-HBV therapeutic target. 

Peng et al. [198] indicated that, in response to HCV core protein stimulation, miR-125b expression was down-regulated in THP-1 cells in contrast to up-regulated cytokine production (e.g., TNF-α, IL-10). Nevertheless, in vitro forced miR-125b expression abolished the HCV core protein-induced enhancement of cytokine expression by targeting TLR2/MyD88 signaling in monocytes, including the phosphorylation of NF-κBp65, extracellular signal-regulated kinases (ERKs), and P38 mitogen-activated protein kinase [198]. Other studies have found that the serum level of miR-125b is increased in HCV infection, in a manner correlated with HCV infection [199,200,201]. These results were confirmed through in vitro studies, showing that the promoter activity and expression of miR-125b were increased in HCV replicon cells, whereas the miR-125b inhibitor reduced HCV expression levels. The results also indicated that the IL-6/STAT3 pathway plays an inducible role in miR-125b expression and may contribute indirectly—through increased expression of miR-125b—to enhanced HCV replication [199]. MiR-125b is involved in translational regulation and may regulate virus replication through human antigen R (HuR), a positive regulator of HCV replication. MiR-125b may also serve as a liver fibrosis biomarker in the context of the viral etiology of HCV [200]. Up-regulated expression of miR-125b has been observed in plasma samples from patients with advanced liver fibrosis in CHC, suggesting that miR-125b has potential as a novel prognostic biomarker, independent of viral replication [201]. Studies have indicated that miR-125b-5p is up-regulated in HCV-infected liver carcinoma cells and down-regulated in exosomes from serum [200,202]. In addition, exosomal miR-125b levels have been used to predict recurrence and survival in HCC patients [202]. 

Hepatitis E virus (HEV) belongs to the family *Hepeviridae*, with an ssRNA genome [203]. It is probably the most common cause of acute viral hepatitis—about 20 million cases are diagnosed annually, and the number of deaths annually has been estimated at 70,000 [204]. Down-regulated miR-125b has been observed in sera from patients with HEV. Interestingly, during acute hepatitis E infection, lower expression of miR-125b was observed compared to samples from patients with chronic hepatis E. Based on these results, researchers have suggested that miR-125b may be a useful biomarker to differentiate acute from chronic viral hepatitis [204].

#### 2.5.2. Respiratory Viruses

Inchley et al. [58] indicated that miR-125b was down-regulated in RVS-infected infants compared to healthy controls [58]; however, based on the results of samples from children with severe RSV-associated pneumonia and samples from mild-RSV-infected children, Zhang et al. [205] found that hsa-miR-125b-5p was significantly increased in samples from children with severe RSV-associated pneumonia. Through gene ontology (GO) enrichment analysis of the target genes of the miRNA, the researchers showed that most target genes were involved in the NF-κB and mitogen-activated protein kinase 1 (MAPK) signaling pathways, which are crucial components of the immune response in humans. Thus, the activation of NF-κB signaling may result in serious complications during severe RSV infection [205]. 

A reduction in miR-125b expression has also been observed in patients infected with H5N1 and H1N1 influenza virus [99,206]. The down-regulation of miR-125b can trigger the MAPK signaling pathway, which regulates various cellular responses, including cell proliferation and apoptosis [206]. 

Interesting observations have been made by Chen et al. [207], who noted that the miR-125b-5p–ACE2–IL-6 axis could alter the risk of SARS-CoV-2 infection in lung adenocarcinoma patients. Reduced miR-125b-5p might be the primary inhibitor of ACE2 in lung adenocarcinoma. Whereas ACE2 was dysregulated, IL-6 in the TLR pathway might activate the immune system as a downstream effector [207].

#### 2.5.3. Human Immunodeficiency Viruses

Another target of miR-125b is cleavage and polyadenylation specificity factor 6 (CPSF6), which plays a key role in HIV-1 infection; specifically, during nuclear import and integration targeting. Researchers reported that HIV-1 infection down-regulated miR-125b expression concurrently with the up-regulation of cleavage and CPSF6, which can contribute to promoting HIV-1 nuclear entry and replication [208]. The inhibitory effect of miR-125b on HIV replication was also demonstrated by Mantri et al. [209], who showed that miR-125b knockdown enhanced HIV-1 replication, whereas the over-expression of miR-125b decreased HIV-1 replication in CD4+ T-cells. The replication control mechanism of HIV may be mediated by targeting of the 3′ UTR regions of HIV-1 transcripts by miR-125b, inhibiting viral protein translation [209]. Additionally, an interesting observation is that miR-125b expression levels are especially high in resting CD4 + T-cells, which may explain the resistance of these cells to HIV-1 infection in comparison to activated CD4 + T-cells, which present down-regulation of miR-125b [110].

#### 2.5.4. Neurotropic Viruses

Japanese encephalitis virus (JEV) belongs to the *Flaviviridae* family, with an RNA genome [210]. Japanese encephalitis virus (JEV) is a neurotropic virus that mainly infects children between 1 and 5 years of age, leading to permanent neuronal damage, motor deficits, and memory loss. The hallmark of JEV is neuroinflammation [211]. MiR-125b, as with other viral infections, plays a significant role in JEV infection. During the infection of cells with JEV, high expression of miR-125b-5p has been observed, which may have an effect on viral replication. These assumptions were confirmed by an in vitro study. The transfection of miR-125b-5p into acutely infected cells reduced the genome replication and virus titers of JEV by targeting STAT3, mitogen-activated protein kinase kinase 7 (Map2k7), and TP53-regulated inhibitor of apoptosis 1 (Triap1). In addition, miR-125b targets the viral genome, suggesting that it could act as a key regulator, providing a balance between viral replication and the host anti-viral response [212].

In summary, the most important action of miR-125b is its participation in viral replication and the immune response.

### 2.6. MicroRNA-132 (miR-132)

MiR-132 is known to control many cellular processes in various tissues, including the modulation of inflammatory processes [213]. It regulates a large number of immune response- and cell cycle-related genes, and may play a role in inhibiting fibrosis through the TGF-β1 signaling pathway [214,215]. MiR-132 also has anti-oxidative stress and anti-apoptotic effects through targeting of the PTEN/Akt pathway [213]. The over-expression of miR-132 induces NF-κB and p65 acetylation [216]. Moreover, miR-132 decreases the production of chemokines (e.g., TNFα, IL-1β) and the capability to attract leukocytes by suppressing the NF-κB pathway [214].

#### 2.6.1. Hepatitis Viruses

Research has shown that miR-132 has a relationship with HBV infection. Interestingly, increased miR-132 expression is influenced by HBx-induced hypermethylation of the DNA promoter [217]. An increase in the expression level of miR-132 can inhibit the expression of some viral proteins, thereby decreasing the expression of anti-viral proteins. However, in liver cancer related to HBV infection, the expression level of miR-132 is down-regulated and its level in serum is significantly correlated with its levels in tumor tissue [217]. Further analysis has revealed that miR-132 exerts tumor-suppressing effects through inactivation of the Akt-signaling pathway, specifically by reducing Akt phosphorylation and the concentration of cyclin D1; therefore, decreased expression of miR-132 may contribute to the development of HBV-related HCC [217]. Liu et al. [218] obtained similar results. They reported significant differences in HBV levels among three groups—CHB, liver cirrhosis, and HCC—in which the HBV-DNA level was highest in the liver cancer group and lowest in the CHB group [218]. Moreover, the level of miR-132 differed remarkably among the three groups, but presented a negative correlation with the HBV-DNA level. In addition, significant differences were detected in the expression levels of genes and protein which regulate cell apoptosis, such as PI3K and p-Akt, in the liver tissues of patients with CHB, liver cirrhosis, and HCC; these, like the level of HBV-DNA, were also negatively correlated with the level of miR-132 [218]. However, further data and more in-depth studies are needed to investigate the role of miR-132 in liver function and the progression of viral liver diseases. 

#### 2.6.2. Respiratory Viruses

Zhang et al. [219] showed that miR-132 was up-regulated in the blood of patients with IAV, and also that IAV infection up-regulated its expression in a dose- and time-dependent manner. Further, an in vitro study in A549 cells indicated that the up-regulation of miR-132-3p promoted IAV replication, whereas the knockdown of miR-132-3p repressed viral replication [219]. Additionally, it was shown that the over-expression of miR-132-3p could inhibit IAV-triggered IFN-α and IFN-β production and ISG expression; however, the suppression of the type I IFN response and the promotion of IAV replication occurred through direct targeting of the miR-132 target gene interferon regulatory factor 1 (IRF1) [219]. Buggele et al. [134] reported that infection with the IAV A/Udorn/72 H3N2 and A/WSN/33 H1N1 strains increased miR-132 expression in the human lung epithelial cell lines A549 and BEAS-2B. Furthermore, miR-132 may regulate innate immune signaling pathways by targeting MAPK3 [134]. Interestingly, miR-132 has been also shown to be regulated transcriptionally through an MAPK3 (ERK1) pathway, and a study on IAV virus has suggested that MAPK3 may use miR-132 to regulate its activity in a negative feedback loop [134]. The expression of miR-132 was also increased in primary bronchial epithelial cells from COPD patients. Enhanced levels of miR-132 decreased the transcriptional coactivator p300, which is essential for the activation of IRF3 and the induction of IFN-β. The up-regulation of miR-132 has been shown to modulate IFN-β induction and impair its function during IAV infection [220]. 

#### 2.6.3. Human Herpesviruses

Herpes simplex viruses (HSVs) type 1 (HSV-1, or human herpesvirus 1—HHV-1) and type 2 (HSV-2, or human herpesvirus 2—HHV-2) are members of the *Herpesviridae* family, with a dsDNA genome [221,222]. Both are closely related, but differ in epidemiology. HSV-1 is associated with orofacial disease and may cause a chronic immuno-inflammatory response in the eye, which is a significant cause of human blindness, whereas HSV-2 is associated with genital disease [222,223]. Mulik et al. [224] showed that miR-132 expression was up-regulated (10-to 20-fold) after ocular infection with HSV-1. This increased miR-132 expression may be caused by VEGF-A and/or IL-17A during infection. The over-expression of miR-132 may lead to the activation of angiogenic Ras, as well as contributing to the immunopathology of stromal keratitis [224]. 

In summary, it can be concluded that the most important role of miR-132, with respect to the considered viral infections, is the regulation of the immune and inflammatory responses.

### 2.7. MicroRNA-34a (miR-34a)

MiR-34a is widely expressed in immune cells (e.g., dendritic cells, macrophages, mast cells, B-cells, and T-cells) and regulates their development, function, and survival. This miRNA, by targeting over 30 genes across different cellular pathways, controls the immune response. MiR-34a expression is controlled through the transcription level of p53 [225]. In addition, miR-34a, through the regulation of Bcl-2, sirtuin 1 (SITR1), cyclin-dependent kinase (CDK) 4, and cyclin D1, among others, induces apoptosis, cell cycle arrest, and/or senescence [226]. MiR-34a plays a significant role in viral infection.

#### 2.7.1. Hepatitis Viruses

HBV infection has a crucial role in cirrhosis and primary liver cancer development. Researchers have shown that miR-34a is down-regulated during HBV infection, and is further lowered in expression in liver cancer [227,228]. HBV, by reducing the level of miR-34a, may modulate the up-regulation of the Wnt/β-catenin pathway through the indirect up-regulation of Wnt1 [229]. In HBV infection, the HBx protein exerts various biological functions related to liver cancer progression, contributing to proliferation, invasion, and venous metastasis [227]. Evidence that HMGB1 (high-mobility group box 1) has enhanced expression in HBV-related liver cancer has been presented, accounting for the epithelial–mesenchymal transition (EMT) and angiogenesis in cancer. A study has proven that HBx-mediated HMGB1 expression is dependent on miR-34a reduction by IL-6/STAT3 [227]. Moreover, it was shown that inhibited miR-34a led to the down-regulation of NF-κB, promoting the expression of HMGB1 and potentially contributing to portal vein tumor thrombus and cancer metastasis [227]. During HBV infection, elevated TGF-β activity in the liver tissue has been observed, which suppresses the expression of miR-34a and leads to the enhanced production of chemokine CCL22 [228,230]. Meanwhile, miR-34a/CCL2 regulation contributes to recruiting regulatory T-cells to facilitate immune escape, favoring the colonization of disseminated HCC cells in the portal venous system [230]. Elevated TGF-β/Smad3 pathway activity and reduced expression of miR-34a also contribute to liver fibrosis in HBV infection, whereas the over-expression of miR-34a in human hepatic stellate cells significantly attenuated fibrosis and TGF-β1/Smad3 activation by targeting Smad4 [228].

Interestingly, during HCV infection, the level of miR-34a is up-regulated. Increased expression of miR-34a has been observed in Huh7.5 HCV-infected cells, as well as in sera from chronic HCV patients [231]. Additionally, miR-34a levels in sera were positively correlated with disease severity, as well as with liver enzyme levels, fibrosis stage, and inflammation activity. Therefore, researchers have postulated that miR-34a may represent a novel, non-invasive biomarker for diagnosis and histological disease-severity determination in patients with CHC [231].

#### 2.7.2. Hemorrhagic Viruses

Rossi et al. [232] have found hsa-miR-34a-5p to be down-regulated in response to DENV-2 infection. Interestingly, miR-34a displays anti-viral activity against flaviviruses, including DENV and WNV [233]. MiR-34a dampens Wnt signaling, allowing the TBK1-mediated phosphorylation of IRF3 in response to pathogen-associated molecular-pattern detection. Moreover, miR-34a may act as a potent activator of the type I IFN response due to the down-regulation of the Wnt/β-catenin signaling members Wnt2 and Wnt3, thereby promoting an anti-viral state [233].

#### 2.7.3. Respiratory Viruses

IAV is a cytolytic virus that induces apoptosis in numerous cell types, and miR-34a is involved in virus-induced apoptosis. A study showed that miR-34a was significantly down-regulated in the sera of infected patients, as well as in IAV-infected A549 cells. Furthermore, an in vitro study indicated that the over-expression of miR-34a could inhibit IV-induced apoptosis by targeting the pro-apoptotic gene Bax [234]. MiR-34 may play a role in IAV infection through regulation of the STAT pathway. Othumpangat et al. [235] demonstrated that, during H9N1 sub-type IAV infection, the level of miR-34 was significantly reduced. Moreover, it was shown that cells transfected with a mimic of miR-34 presented modulated phosphorylation and decreased expression of STAT3, which plays a critical role in anti-inflammatory function and inhibits NF-κB gene reporters. In addition, STAT3, by inhibiting NF-κB, may inhibit IAV replication [235].

MiR-34a has also been found to be down-regulated in the lung tissues of COVID-19 patients. In silico analysis indicated that miR-34a may have an effect on apoptosis through Bcl-2, BCL2 associated X (Bax), and Kruppel-Like Factor 4 (KLF4) genes, as well as on inflammation through the interleukin 6 receptor (IL-6R) [236]. However, further research showed that in lung tissues, only CASP-1 (caspase 1)—which is involved in the signaling pathways of apoptosis, necrosis, and inflammation—underwent increased expression, and was negatively correlated with miR-34a levels [236]. Additionally, a reduction in host miR-34a-3p in COVID-19 could increase X-box-binding protein 1 (XBP1s) expression by URP, increasing the ER folding capacity, inhibiting lung fibrosis, and protecting against over-activation of the immune system, thus promoting survival [237]. There have also been reports that miR-34a, along with other miRNAs, may target the membrane (M) protein gene of SARS-CoV-2, defining the shape of the viral envelope and the central organizer of CoV assembly [238].

#### 2.7.4. Human T-Lymphotropic Virus Type 1

Human T-lymphotropic virus type 1 (HTLV-1) is a member of the *Retroviridae* family, with an ssRNA genome [109]. The virus can cause a type of cancer called adult T-cell leukemia/lymphoma [239]. Sharma et al. [240] showed that infected cell lines expressed higher levels of miR-34a compared to normal PBMC or purified CD4+ T-cells. Further analysis indicated that the primary miR-34a transcript contained binding motifs for NF-κB and p53. Moreover, the treatment of infected cell lines with the p53 activator resulted in a further increase in miR-34a levels [240]. This increase in miR-34a contributed to the down-regulation of the deacetylase SIRT1, as well as the pro-apoptotic factor Bax. These findings suggest a functional role for miR-34a in fine-tuning the expression of target genes which influence the turnover of HTLV-1-infected cells [240].

Our review indicates that the most important role of miR-34a in the considered viral infections is modulating the immune response and the course of apoptosis.

### 2.8. MicroRNA-21 (miR-21)

MiR-21 plays a crucial role in many biological functions and diseases, including development, cancer, cardiovascular diseases, and inflammation [241]. The down-regulation of miR-21 increases the rate of cell death, most probably by targeting HIF-1α, phosphatase and tensin homolog (PTEN), and programmed cell death 4 (PDCD4). Meanwhile, the upregulation of miR-21 by cytokines during most viral infections indicates it role in inflammation, and may lead to host immune system dysfunction and viral replication [242]. MiR-21 has also been accepted as an activator of regeneration processes in tissue damage repair [243].

#### 2.8.1. Hepatitis Viruses

Liver fibrosis has been considered as a healing response to various chronic liver injuries, including viral hepatitis [244]. Chronic HBV infection is a major risk factor for HCC, which is one of the most common cancers worldwide [245]. HBx protein is known to be involved in the initiation and progression of HCC through the modulation of the host gene response [246,247]. Wu et al. [248] demonstrated that miR-21 was expressed in the sera of patients with CHB. In addition, the expression level of miR-21 was significantly correlated with the histological stage of liver fibrosis and cirrhosis [248,249]. Studies have shown that miR-21 activates hematopoietic stem cells (HSCs) in the liver through PTEN/Akt signaling, and may also promote α-SMA and collagen I expression in HSCs through the Smad 7 signaling pathway [250,251]. Another study has shown that miR-21 may play a role in liver fibrosis with HBV etiology, mediated via transforming growth factor beta 1 (TGF-β1) signaling [249]. HBV infection up-regulated TGF-β1/miR-21-5p mRNA expression in NTCP-Huh7.5.1 cells. Cells incubated with TGF-β1 presented significantly increased miR-21-5p levels, as well as the mRNA and protein expression of α-smooth muscle actin (α-SMA), collagen type 1 α1 (CoL1A1), and tissue inhibitor of metalloproteinase 1 (TIMP-1), along with reduced Smad7 expression in human hepatic stellate (LX2) cells. Interestingly, the over-expression of miR-21 in LX2 cells can cause a positive feedback loop and up-regulate TGF-β1 activation in cells, contributing to fibrosis in HBV infection [249]. Researchers have noted the role of miR-21 in carcinogenesis caused by HBV infection [252,253,254,255,256,257]. Qiu et al. [252] indicated that HBx down-regulated PDCD4 through the up-regulation of miR-21 expression. The over-expression of HBx in Huh.7 and HepG2 cells enhanced miR-21 expression. The up-regulation of miR-21 can increase proliferation and decrease target proapoptotic protein (PDCD4 and PTEN) expression, as well as activating Akt [253]. PDCD4 and PTEN have strong tumor-suppressive effects both in vitro and in vivo, and may induce cell apoptosis to suppress the development of HCC; however, their suppression by miR-21 may lead to the progression of HCC [252,253]. Another mechanism of miR-21 regulation by HBx and subsequent HCC development is mediated by the HBx-induced interleukin-6 pathway followed by activation of the STAT3 transcriptional factor. The induction of miR-21 by the IL-6/STAT3 pathway is essential for transforming non-tumor hepatocytes, implying a critical role in early HCC development during chronic HBV infection [254,255]. Yin et al. [257] have additionally shown that IL-12 is a direct target of miR-21 in HBV infection and HCC growth. IL-12 promotes the effective destruction of cancer cells by inducing the proliferation of NK and T-cells, and enhances the generation and activity of cytotoxic T-lymphocytes [258,259]. In an in vitro study, the inhibition of miR-21 resulted in a significant increase in apoptosis and increased IL-12 expression. Therefore, the results suggest that HCC cell apoptosis was suppressed—at least partially—through HBx-induced miR-21 targeting IL-12 [257]. 

In hepatic tissues from HCV-infected patients, the expression of miR-21 has also been found to be increased [260,261,262]. Additionally, in Huh.7 cells, the transduction of the HCV-3a core up-regulated miR-21 expression and/or its activity. Enhanced miR-21-5p bioavailability and binding to specific targets, such as PTEN, and its down-regulation affect lipid accumulation in Huh-7 cells. Additionally, research showed that the genetic deletion of miR-21-5p in mice reduced HCV-3a-induced steatosis in vivo, suggesting that miR-21-5p activation/up-regulation may represent a key event in the pathogenesis of steatosis-associated oncogenesis [260]. Interestingly, miR-21-5p may also promote HCV replication and increased virion production [260,261]. The up-regulation of miR-21 by HCV suppresses HCV-triggered type I IFN production, thus promoting HCV replication [261]. Furthermore, Chen et al. [261] have identified a virus–host interaction pathway, in which HCV infection results in the stimulation of two signaling pathways: The NS5A/protein kinase C (PKC) ε/c-Jun N-terminal (JNK)/c-Jun pathway and the NS3/4A/PKCα/ERK/c-Fos pathway. After infection, c-Jun and c-Fos bind to the AP-1 binding sites in the miR-21 promoter and mediate the induction of miR-21. The induced miR-21 targets two important factors in the TLR signaling pathway—myeloid MyD88 and IRAK1—which are involved in HCV-induced type I IFN production. The silencing of MyD88 and IRAK1 by miR-21 and negative regulation of type I IFN may be potential therapeutic targets for anti-viral intervention [261]. Similarly to HBV, in HCV infection, miR-21 has been found to be correlated with fibrosis stage and, by targeting SMAD7, could increase TGF-β signaling, leading to increased fibrogenesis. Moreover, in HCV infection, miR-21 was also correlated with viral load and serum liver transaminase levels, making it a good candidate as a biomarker [262].

#### 2.8.2. Hemorrhagic Viruses

MiR-21 may also serve as a biomarker in DENV infection. Ouyang et al. [132] have indicated that miR-21 is up-regulated in the course of dengue, and could be used to distinguish dengue-infected patients with preferable sensitivity and specificity. In addition, they showed that miR-21 had a positive correlation with serum AST and ALT activities, and negative correlations with WBC, platelet (PLT), neutrophil, and lymphocyte numbers [132]. Other researchers have noted the role of miR-21 in dengue virus replication. The results showed a significant reduction in DENV 2 production in HepG2 cells after treatment with an anti-miR-21 (AMO-21), clearly suggesting that miR-21 plays a key role in DENV 2 replication [263]. Furthermore, in hemorrhagic fever caused by Ebola, upregulation of miR-21 was also observed [264].

#### 2.8.3. Respiratory Viruses

In response to influenza A virus (IAV) infection, a wide range of innate immune factors and miRNAs, such as miR-21, may be involved in controlling acute influenza. Down-regulation of miR-21 has been observed in the sera of H5N1-infected patients and in A549-infected cells [265,266]. In infected cells, the level of miR-21-3p was conspicuously down-regulated with prolonged infection. H5N1-infected A549 cells transfected with mimic-21-3p contributed to the augmentation of infectious progeny virions, whereas infectious virions were effectively decreased in the inhibitor-21-3p group. Additionally, researchers reported that mimic-21-3p dramatically down-regulated the levels of IFN-β and IFN-α in infected cells, while the silencing of miR-21-3p produced the opposite results [265]. Moreover, this miRNA has been shown to be involved in the NF-κB signaling pathway, and NF-κB affects the production of a type I IFN response [267]. Thus, miR-21 may affect IAV replication. Shi et al. [265] have demonstrated that miR-21, by targeting FGF2, can accelerate H5N1 replication in H5N1-infected A549 cells by inhibiting the type I IFN response [265]. Another study has shown that promoting IAV replication by miR-21 may take place also through histone deacetylase-8 (HDAC8) [266]. Lam et al. [268] showed that influenza H5N1 and H1N1 infection down-regulated miR-21 expression. Interestingly, the researchers noted that, in H1N1 infection, miR-21 was increased 24 h after infection. Research using the TargetScan software also indicated that miR-21 may regulate CCL1 (small inducible cytokine A1 precursor), CCL17, CCL19, IL22, apoptosis-related protein 3 precursor (C2orf28), and tumor necrosis factor ligand superfamily member 12 (TNFSF13) in the context of influenza infection [268]. Meanwhile, the elevated miR-21 level on day 15 of IAV infection was identified to be important in the late repair phase through the targeting of proliferation-suppressing factors [269,270]. In addition, its elevated expression during repair coincides with increased proliferation in repairing lungs. Notably, increased miR-21 levels may lead to detrimental effects, such as fibrosis, through targets in the TGF-β pathway [271]. 

MiR-21 has also been shown to be down-regulated in adenovirus type 2 infection of human lung fibroblasts. The researchers showed that its expression was also the most strongly down-regulated (more than 9-fold) 12 h after infection, and that it may target an important cellular network of tumor-suppressor genes, such as p53, TGF-β, and mitochondrial apoptosis genes [80]. 

In contrast to the main trend of decreased miR-21 expression in IAV infection, in SARS-CoV-2-infected patients, significantly up-regulated miR-21 levels have been observed, which may be fibrosis-associated [76,272]. The up-regulation of miR-21 may also serve as a predictor of chronic myocardial damage and inflammation. Interestingly, the level of miR-21 was decreased in patients who died due to COVID-19, and was also correlated with a higher rate of extracorporeal membrane oxygenation (ECMO) and renal replacement therapy [76]. Researchers have shown that miR-21-3p can bind sites on the open reading frame (ORF) 1ab, ORF3a, and spike of SARS-CoV-2, which encodes the spike protein necessary for viral entry and is a promising target for anti-viral therapy. In connection with the impact of miR-21-3p, host and virus genome experimental validation of miR-21’s involvement in both binding to the SARS-CoV-2 genome and modulating the host transcriptome have been suggested [273]. During COVID-19, miR-21-5p may directly target CCL20, which is up-regulated in the inflamed airway epithelium, as well as MYC, the over-expression of which fosters the inflammatory response and T-cell metabolic reprogramming, respectively [139]. Other targets for miR-21 are IRAK1, which participates in the NF-κB pro-inflammatory signaling pathway, as well as CXCL-10 [139,273], a biomarker for viral infection. Increased levels of this chemokine have been observed in the lungs of COVID-19-infected patients compared to healthy ones [273]. 

In RSV-infected cells, significant up-regulation in the composition of exosome miR-21 has been observed [274,275]. Exosomes released from virus-infected A549 cells can alter innate immune responses through the induction of pro-inflammatory mediators. Antagonistic miR-21 treatment inhibited eosinophil inflammation and AHR in an RSV-induced steroid-insensitive mouse airway allergic-disease model [276]. Therefore, miR-21 may be a key signal regulating the balance and transition between pro-inflammatory and immune activation [277].

#### 2.8.4. Human Immunodeficiency Viruses

In HIV patients, a low level of exosomal miR-21 in the plasma, along with a decrease in CD4+ T-cells, was observed [278]. In Jurkat cells, the stable intracellular expression of Tat (HIV gene) induced an increase in miR-21 expression, which led to apoptosis- and cell cycle-related proteins being down-regulated, including PTEN, PDCD4, and cyclin-dependent kinase inhibitor 1B (CDKN1B) [279]. It has been further demonstrated that the over-expression of miR-21 significantly inhibits IP-10, a key inflammatory cytokine that causes immune dysfunction and facilitates HIV infection [280].

#### 2.8.5. Cardiotropic Viruses

Coxsackievirus B3 (CVB3) belongs to the *Picornaviridae* family, with an RNA genome [83]. CVB3 is a common and important pathogen of viral myocarditis, pancreatitis, and aseptic meningitis in young children and infants. CVB3 infection may lead to acute heart failure and sudden death due to direct cytopathic effects induced by viral replication in the early phase of infection [281,282]. Similar to DENV, miR-21 plays an important role in viral replication during CVB3 infection. In CVB3-infected HeLa cells, significant up-regulation of miR-21 expression, followed by the suppression of mitogen-activated protein kinase kinase 3 (MAP2K3)/P38 MAPK signaling, has been observed. Furthermore, miR-21 over-expression significantly inhibited the release of virions from CVB3-infected cells. This decreased viral release was accompanied by a significantly alleviated myocyte cell apoptosis rate, reduced viral titers, lower necrosis in the heart, and remarkably prolonged survival time in an in vivo study [283]. Ye et al. [284] have indicated that over-expressed miR-21 during CVB3 infection may target deubiquitinating enzyme (YOD1) to enhance the lysine 48-linked ubiquitination and degradation of desmin; this results in the disruption of desmosomes, which are substantial connections maintaining cardiac structures and mediating signal communications among cardiomyocytes. In addition, miR-21 directly targets vinculin, leading to disturbed fascia adherens, as evidenced by the suppression and disorientation of pan-cadherin and α-E-catenin proteins, two fascia adherens components. Thus, miR-21 may contribute to the pathogenesis of viral myocarditis [284].

#### 2.8.6. Vector-Borne Viruses

Chandipura virus (CHPV) belongs to the *Rhabdoviridae* family, with an RNA genome [285]. It causes acute encephalitis with a case fatality rate of 70%. An in vitro study has indicated over-expression of miR-21 in human microglial cells infected with CHPV. The higher level of miR-21 leads to the down-regulation of PTEN, which promotes the phosphorylation of AKT and NF-ĸB (p65). Moreover, the activation of NF-ĸB increases the transcription of pro-inflammatory cytokines (e.g., IL-6 and TNF-α) [286].

In conclusion, invalid miR-21 expression is closely associated with viral infections, where increased levels of miR-21 may enhance viral replication. In addition, research has shown that miR-21 may be involved in organ damage associated with active viral infection. As such, miR-21 has increasingly been described as a potential biomarker of infection.

### 2.9. MicroRNA-16 (miR-16)

MiR-16 can modulate the cell cycle, inhibit cell proliferation, and promote cell apoptosis [287]. These effects can be explained with respect to the targeting of miR-16 in the anti-apoptotic gene Bcl-2 (B-cell lymphoma 2); numerous genes involved in the G1-S transition, such as cyclin D1, cyclin D3, cyclin E1, and CDK6 (cyclin-dependent kinase 6); and genes involved in the Wnt signaling pathway, such as WNT3A (wingless-type MMTV integration site family member 3A) [288]. Additionally, miR-16 directly targets PDCD4 to suppress the activation of inflammatory macrophages through the mitogen-activated protein kinase (MAPK) and NF-κB pathways. In these ways, miR-16 contributes to decreasing the levels of inflammatory cytokines (e.g., IL-6, TNF-α, and IFN-β), while simultaneously enhancing the secretion and mRNA expression of the anti-inflammatory factor IL-10 [289].

#### 2.9.1. Hepatitis Viruses

MiR-16 may a play a significant role in infection with hepatotropic viruses and fibrosis. Wu et al. [290] indicated that the HBx protein decreased the expression of miR-16 in host malignant hepatocytes (i.e., human HepG2, SK-HEP-1, and Huh7 HCC cell lines) in vitro. They also examined the expression of target genes for miR-16, indicating that cyclin D1 (CCND1)—which functions in the G1/S transition of the cell cycle—was significantly up-regulated [290]. Furthermore, the HBx-induced down-regulation of HepG2 cells was c-Myc mediated. Meanwhile, ectopically expressed miR-16 repressed the proliferation, clonogenicity, and growth of HepG2-hbx cells by inducing cell cycle arrest (CCND1), and apoptosis by targeting Bcl-2 [290]. However, further studies are needed in order to clarify the roles played by miR-16 in HBx-induced hepatocyte apoptosis (or the inhibition of apoptosis) and acute/chronic HBV infection. 

HCV is involved in the initiation and progression of liver fibrosis by miRNA and regulating genes encoding host proteins. It was observed that miR-16 levels were increased in the PBMCs and sera of patients with chronic HCV and in liver cell lines infected with HCV. Additionally, the miR-16 level was negatively correlated with HGF and Smad7 expression [291]. This suggests that miR-16 may contribute to the development of liver fibrosis. Interestingly, a study showed that the treatment of HCV- cells infected with IFN-α led to the down-regulation of miR-16 and up-regulation of hepatocyte growth factor (HGF) and Smad7 [291]. MiR-16 has also been shown to be correlated with ALT and AST levels, but not with the stage of fibrosis [231,291].

#### 2.9.2. Respiratory Viruses

The cellular response to viral infection is initiated by recognition of the invading pathogen and subsequent changes in gene expression mediated by both transcriptional and translational mechanisms. Buggele et al. [134] showed that the expression of miR-16 during IAV infection in primary airway epithelial cells increased 8 h after infection, while it was significantly decreased at 24 h [134]. In addition, the miR-16 level was negatively correlated with that of IFN-β. Interestingly, in A549 and BEAS-2B cells infected with A/Udorn/72 and A/WSN/33 strains of influenza virus, miR-16 presented constant expression levels throughout the infection [134]. Research has also demonstrated that NS1 in a specific strain of IAV may contribute to the translocation of miR-16 and AGO2 from the cytoplasm to the nucleus, thus enhancing the in vivo virulence of IAV [292]. 

In silico analysis has indicated that miR-16 may bind, with high probability, to the RNAs of the human coronaviruses MERS-CoV, SARS-CoV, and SARS-CoV-2. Interestingly, most binding sites were found within NS proteins located in the polyprotein 1ab coding region [272]. During SARS-CoV-2 infection, miR-16 was predicted to target the largest number of differentially expressed host genes. Given that the majority of miR-16 targets, including mitogen-activated protein kinase-activated protein kinase 2 (MK2) and CCND1, were down-regulated in response to SARS-CoV-2, the expression of miR-16 may be increased in the lungs of COVID-19 patients [273]. 

In children with RSV infection, miR-16 has been shown to be significantly up-regulated [58]. It may have an effect on the NF-κB pathway, which is activated following RSV-antigen binding to the pathogen recognition receptors TLR 4 or RIG-1, as a primary stage in the immunological response to RSV. However, excessive NF-κB activation may also have deleterious effects and, so, negative regulation is also important. Therefore, miR-16 may have a significant role related to the consistent fine-tuning of the immune response to RSV [58]. 

Our review demonstrates that the most important function of miR-16 is the regulation of hepatocyte apoptosis in infection with hepatotropic viruses, as well as the fine-tuning of the immune response in the course of infection with respiratory viruses.

### 2.10. MicroRNA-181 Family (miR-181)

The miRNA family miR-181 has diverse roles, in terms of regulating key aspects of cellular growth, development, and activation. By regulating critical signaling pathways, such as NF-κB, the miR-181 family plays a key role in inflammation [293]. Moreover, it targets importin-α3, a protein critical for NF-κB nuclear translocation, and negatively regulates pro-inflammatory cytokines [294]. The miR-181 family also regulates apoptosis by directly targeting relevant genes [295,296]. Therefore, increasing attention has been paid to the implications of the miR-181 family in the field of immunology and viral disease.

#### 2.10.1. Hepatitis Viruses

The miR-181 family plays a significant role in hepatitis virus, and may also be a potential marker in hepatitis diagnostic and liver failure. Yu et al. [297] demonstrated that miR-181 levels were up-regulated and correlated with liver and serum HBV DNA levels and disease progression. Additionally, the serum miR-181b level was associated with the fibrosis score, suggesting that miR181b acts as a pro-fibrosis miRNA in the liver and, so, has potential as a marker for disease progression in CHB patients [297]. Studies have shown that miR-181b promotes hepatic stellate cell proliferation by targeting the p27 and PTEN/Akt pathways, and is elevated in the sera of cirrhosis patients, further indicating its role as a pro-fibrotic factor [298,299]. Another miR from the miR-181 family which targets PTEN is miR-181a. In vitro research indicated that HBV (HBx protein) increased the expression of miR-181a [300,301,302], which then reduced PTEN protein expression [300]. The miR-181a/PTEN pathway during HBV infection contributes to cell proliferation and suppresses apoptosis, leading to the development of hepatocellular carcinoma [300]. Another mechanism of miR-181a affecting HCC formation is that the inhibition of the expression of transcription promotes tumor cell growth in vivo through the suppression of TNF receptor superfamily member 6 (Fas) expression in hepatoma cells [302]. These data indicate that miR-181a plays an essential role in the regulation of HCC cell proliferation, and may function as an onco-miRNA in HBV-related HCC [300,301,302].

In HCV infection, researchers have noted the up-regulation of miR-181a in infected patients [303], as well as its down-regulation in liver tissues [304]. Additionally, serum miR-181a expression in HCV patients is inversely correlated with the level of viremia, as well as liver enzymes (ALT and AST). They also observed significant up-regulation of miR-181a serum levels in interferon-treated patients who had developed a sustained virological response. However, Elhelw et al. [303] did not observe a difference in miR-181a expression in the liver tissues of patients, compared to controls [303]. MiR-181a was also down-regulated in liver fibrosis stages 2 and 3 caused by HCV infection. In addition, miR-181a was negatively correlated with the grade of necroinflammatory activity during HCV genotype 2 infection. An analysis of different types of necroinflammatory activity demonstrated the expression of miR-181a to be involved in periportal/periseptal inflammation [304]. HCV infection also decreased the expression of miR-181a in CD4+ T-cells, where the decline in miR-181a expression impaired CD4+ T-cell responses via the over-expression of dual specific phosphatase 6 (DUSP6) [305]. In particular, a significant decline in miR-181a expression, along with the over-expression of DUSP6, was observed in CD4+ T-cells from chronically HCV-infected individuals. However, treatment with miR-181a precursors in CD4+ T-cells led to improved T-cell responses and increased expression of IL-2 [305] and factor E2F5, which is a key regulator of cell growth [301]. During HBV infection, miR-181a inhibits apoptosis in vitro.

These data suggest that miR-181a may be considered to be a possible prognostic marker in HCV infection [303,304,305]. Another member of the miR-181 family that is decreased in HCV is miR-181c [305,306]. Mukherjee et al. [306] observed that HCV infection of hepatocytes transcriptionally down-regulated miR-181c expression by modulating C/EBP-β. The reduction in miR-181c levels led to enhanced expression of HOXA1 (homeobox A1), a cell growth regulator that can enhance oncogenic transformation through STAT3 and STAT5 [306]. Cell transfection with an miR-181c mimic contributed to the downregulation of HOXA1, potentially reducing the risk of developing HCC. Interestingly, the over-expression of miR-181c also inhibited HCV replication through direct binding with the E1 and NS5A sequences [306]. Another direct target of miR-181c is ATM (ataxia-telangiectasia mutated). A study demonstrated that ATM expression was higher in HCV-infected hepatocytes and chronic HCV-infected liver biopsy specimens [307]. The exogenous expression of miR-181c inhibited ATM expression and the activation of its downstream molecules, Chk2 (checkpoint kinase 2) and Akt. Furthermore, the over-expression of miR-181c significantly inhibited phospho-CDK2 and Cyclin-A expression, arresting cell cycle progression while simultaneously promoting the apoptosis of HCV-infected hepatocytes [307]. Considering the presented data, miR-181c seems to be an important factor in HCV–hepatocyte interactions, and may serve as a target for therapeutic intervention [306,307].

#### 2.10.2. Respiratory Viruses

Researchers reported that a member of the miR-181 family, miR-181c, was up-regulated in A549 cells infected with H5N1, H3N2, and H1N1 influenza A viruses [206]. In addition, the increase in the level of this miRNA leads to the targeting of many genes associated with cellular immune defense, such as BCL2, IL-2, and TNF-α [206]. Researchers are increasingly focusing on miRNAs as potential biomarkers of infectious diseases. Lim et al. [308] have identified miR-181c-5p as a potential biomarker for the detection of pandemic influenza A H1N1 virus infection [308]. Moreover, miR-181a-5p has been selected as a biomarker to differentiate IAV or influenza B virus (IBV) patients from healthy controls. Furthermore, it is useful for the diagnosis of H1N1 and H3N2 infection. However, in contrast to miR-181c, influenza A virus down-regulates the level of miR-181a in infected patients [309].

Interestingly, during RSV1 and RSV3 infection, the expression of miR-181a is up-regulated [178]. Furthermore, the vaccination of mice with a live attenuated candidate for RSV resulted in increased miR-181a levels [310]. Therefore, these results suggest that miR-181a may have a significant role in the response to RSV infection [178,310]. 

In viral infections, members of the miR-181 family regulate many genes related to the cellular immune response. The most important aspect of this family is the use of these molecules as potential biomarkers for the diagnosis of viral infections due to their good correlations with viral load levels and various markers (e.g., liver enzymes).

### 2.11. Let-7 Family (Let-7)

Let-7 miRNA was first discovered in *Caenorhabditis elegans*, and is highly conserved in human tissues. The human let-7 family of miRNA contains 12 members [311]. The let-7 family is involved in many biological processes. Studies have revealed the role of let-7 members in the regulation of the cell cycle, proliferation, and apoptosis and, above all, in the processes of oncogenesis [311,312]. The let-7 family has also been implicated in the post-transcriptional control of innate immune responses to various pathogenic agents, including viruses [313,314].

#### 2.11.1. Hepatitis Viruses

The Let-7 family is frequently down-regulated in multiple human tumors, including hepatocellular carcinoma (HCC), caused (inter alia) by chronic infection with HBV and HCV. Studies have shown that the HBx protein down-regulates the entire let-7 family in HepG2 line cells [315,316]. In addition, it has been indicated that let-7a negatively regulates cellular proliferation, partly through the targeting of STAT3, which is involved in many cellular processes (including cell growth, survival, metastasis, angiogenesis, and immune suppression), all of which favor tumor formation and progression [315,317]. Therefore, the downregulation of let-7 by HBx supports cell proliferation and may contribute to HCC [315]. The lowered let-7 levels in HBV infection may be caused by the over-expression of Lin-28 Homolog B (Lin28B). Lin28B is over-expressed in HBx-transfected cells and HBV-infected liver tissues, and the HBx–c-Myc–Lin-28 homolog B (Lin28B) axis was found to mediate the repression of let-7 in HepG2 cells [316]. Interestingly, Deng et al. [194] have discussed the implications of Lin28B/let-7 and miR-125b in HBV infection. They demonstrated that miR-125b-5p targets the Lin28B/let-7 axis and contributes to the down-regulation of lin28B and up-regulation of let-7 in order to stimulate HBV replication in a post-transcriptional step [194]. Qiu et al. [318] have shown that the level of let-7a was lower in malignant tissues than in adjacent normal tissues; however, patients with highly active HBV replication demonstrated a significantly higher level of let-7a in hepatocarcinoma tissue than patients with less-active HBV replication [318]. In addition, an in vitro study demonstrated that the down-regulation of let-7a by anti-sense oligonucleotides led to a reduction in HBV DNA copy numbers. This indicated a correlation between the let-7a level and HBV replication, suggesting that the down-regulation of let-7a reduces HBV replication and could prevent the development of HCC [318]. Furthermore, let-7g may affect HBV replication. In an in vitro study, Takata et al. [319] showed that cells over-expressing let-7g presented suppressive effects on replication and HBV protein levels. However, HBV mRNA in the surface protein preS2 region can sequester let-7g which, in turn, impairs the intrinsic let-7g function and is crucial in the pathogenesis of chronic viral infection [319].

Similarly as in HBV infection, in HCV, the down-regulation of let-7a and let-7b was also observed in tissue, particularly, during HCV infection and in HCV-infected cell culturing. However, an in vitro study has shown the function of let-7 in terms of restricting multiple steps of the HCV life cycle; namely, entry, translation, and RNA replication. The over-expression of let-7a in Huh.7.5.1 cells significantly reduced HCV core production expression, HCV RNA production, and viral infectivity, confirming an anti-viral role of let-7a in hepatocytes. Let-7a targets component of inhibitor of nuclear factor kappa B kinase complex (CHUK), inhibitor of nuclear factor kappa b kinase subunit epsilon (IKBKE), and X-prolyl aminopeptidase 1 (XPNPEP1), preferentially acting on HCV assembly or secretion; this implies that let-7a also acts in the late stage of the HCV life-cycle. In addition, let-7a also targets CLDN1 and represses its translation to block HCV entry [320]. Another observation, made by Cheng et al. [321], indicated that let-7b was induced during the early stage of HCV infection and, similarly to let-7a, suppressed HCV replication. Their data suggest that let-7b suppressed HCV replicon activity and down-regulated HCV accumulation, leading to reduced infectivity by binding to the coding sequences of NS5B and the 5′-UTR of the HCV genome. However, the mechanism for the let-7b-mediated suppression of HCV RNA accumulation was not dependent on the inhibition of HCV translation [321]. Another study has shown that let-7b directly targets negative regulators of type I IFN signaling, thereby limiting HCV replication in the early stage of HCV infection [322]. Additionally, let-7b targets SOCS1, leading to the increased expression of downstream ISGs. On the other hand, let-7b directly targets the ATG12 and IκB kinase alpha (IKKα) transcripts and reduces the interaction of the ATG5–ATG12 conjugate with RIG-I, leading to the increased expression of IFN, which may then stimulate JAK/STAT signaling [322]. Studies have also shown that let-7b can affect HCV replication by binding and, thereby, reducing the expression of the factor insulin-like growth factor 2 mRNA-binding protein 1 (IGF2BP1), which is required for HCV replication [323]. In addition to changes in the expression of miRNA in tissue samples or in cell cultures, miRNAs in the let-7 family also present changes in sera. Studies have shown that, along with the severity of the disease, decreased levels of let-7a/7c/7d-5p have been observed in the sera of HCV patients. In addition, levels of let-7 family members have been correlated with the advanced histological hepatic fibrosis stage and other fibrotic markers, including Mac-2-binding protein glycan isomer (M2BPGi), fibrosis-4 (FIB-4) index, and the aspartate aminotransferase-to-platelet ratio index (APRI) [324,325]. Furthermore, pathway analysis has suggested that low levels of let-7 may influence hepatic fibrogenesis through the activation of TGF-β signaling in hepatic stellate cells [325]. 

#### 2.11.2. Hemorrhagic Viruses

In an in vitro study, a role of let-7c in DENV infection has been also demonstrated. During dengue infection (DENV2 and DENV4) of the liver culture cell Huh.7, as well as in the macrophage–monocytic cell line U937-DC-SIGN, the over-expression of let-7c was observed [326]. As with other infections, let-7 family miRNA—more specifically, let-7c—inhibited DENV infection. Escalera-Cueto et al. [326] have demonstrated that let-7c inhibits infection with DENV2 and DENV4 through the down-regulation of Bach1 and over-expression of HO-1, which is caused by Bach1 depression. The indirect up-regulation of HO-1 by let-7c induces anti-oxidative and anti-inflammatory responses, which may be necessary to counteract harmful effects in infected tissues [326].

#### 2.11.3. Respiratory Viruses

The up-regulation of pro-inflammatory factors usually activates the expression of anti-inflammatory factors and causes homeostasis responses to inflammatory stress during viral infection [327]. The homeostasis response may be mediated by both mRNA and miRNA. Let-7 is a type of miRNA that binds to the 3′-untranslated region of IL-6 mRNA and inhibits pro-inflammatory IL-6 mRNA and protein expression. Zhang et al. [327] demonstrated that let-7e was significantly increased in THP-1 cells treated with hemagglutinin (HA) protein of the avian influenza A (H7N9) virus. However, HA may inhibit the secretion of let-7e from THP-1 cells by activating the TLR4 pathway, and can enable the maintenance of high intracellular let-7e levels [327]. Interestingly, Zhu et al. [328] observed that patients infected with H7N9 showed decreased let-7e levels in sera, indicating that the cells of these patients attempted to trigger a protective response to avoid severe inflammatory damage in specific organs or tissues [328]. Another miR from the let-7 family also plays a role in protecting host cells from the virus: it was observed that let-7c was highly up-regulated in influenza virus-infected human lung epithelial cells (A549). This study also showed that let-7c directly targeted the 3′-UTR of the IAV matrix protein (M1) and affected the reduction of M1 levels in A549 cells at the cRNA and protein levels, where the decrease in M1 protein caused a reduction in IAV replication [329]. Interestingly, let-7c was also up-regulated within 15 days of infection with IAV (H1N1) in murine lung tissue. TargetScan analyses also revealed that let-7c is involved in targeting relevant gene functions in repair, thus limiting damage and accelerating repair after infection [269]. 

In RSV-infected A549 cells, let-7f is up-regulated by the RSV G protein. The results of this study also showed that let-7f regulates CCL7/MCP-3 and SOCS3, which are involved in the anti-viral cytokine response (e.g., the type I IFN response) and regulate ELF4—a known inducer of IL-8 in RSV infection [330]. Furthermore, up-regulation of let-7d has been detected in epithelial cells of the nasal mucosa in children with RSV infection [58]. Thornburg et al. [331] have demonstrated that let-7b is up-regulated in DCs, while let-7i is up-regulated in epithelial cells in a process that requires viral replication. Interestingly, researchers found that the RSV non-structural genes NS1 and NS2 antagonized the up-regulation of let-7i. In addition, they indicated that the induction of let-7b and let-7i was also enhanced by IFN-β [331]. In contrast to the above-mentioned studies, in Calu3 respiratory cells infected with RSV, let-7f was down-regulated, accompanied by the up-regulation of IFN-λ, which could induce innate anti-viral responses in epithelial cells [332]. 

As in the previously discussed respiratory viruses, in the course of COVID-19, the up-regulation of let-7b was obviously observed in PBMCs. In patients with post-acute COVID-19, a higher level of let-7b expression occurs compared to acute infection; this potentially suggests a role of let-7b in the immune response and repair function through the targeting of genes such as V-Rel avian reticuloendotheliosis viral oncogene homolog B (RELB), IL-6, KH-type splicing regulatory protein (KHSRP), euchromatic histone lysine methyltransferase 2 (EHMT2), lysine demethylase 2B (KDM2B), C-MYC, and LIN28B [71]. The over-expression of let-7b has also been observed in neutrophils during SARS-CoV-2 infection, which could alter their function by suppressing the TLR4/NF-κB signaling pathway [333]. Moreover, Chen et al. [333] have demonstrated, through an in vitro study, that the up-regulation of let-7b may decrease the levels of various pro-inflammatory factors, including IL-6, IL-8, and TNF-α, and up-regulate the anti-inflammatory factor IL-10. This study suggested that let-7b could reduce complications associated with COVID-19, such as viral sepsis [333]. Additionally, as in IAV infection, another miRNA from let-7 family—let-7c—may have an effect on the replication of SARS-CoV-2 by targeting ORF1ab, encoding the 5′-viral replicase [238]. An experimental study also showed that let-7d, 7e, 7f, 7g, and 7i were able to significantly suppress the expression of the S protein, while let-7b, 7c, 7g, and 7i inhibited M protein expression, thereby blocking SARS-CoV-2 replication. The researchers also showed that let-7 suppresses the expression of multiple inflammatory factors, including IL-1β, IL-6, IL-8, CCL2, GM-CSF, TNF-α, and VEGFα, by targeting the IL-6/STAT3 pathway, leading to a reduction in inflammation [334]. Interesting observations have been made by Chow et al. [74], who reported that differential expression analysis of Calu3 cells infected with SARS-CoV-2 revealed that hsa-let-7a-3p was down-regulated, in contrast to other considered miRs from the let-7 family [74].

#### 2.11.4. Human Immunodeficiency Viruses

Swaminathan et al. [335] have shown that, in infected HIV-1 cells, let-7 can repress IL-10 expression at the post-transcriptional level. They found that IL-10 was highly upregulated in HUT78 T-cells, and proposed that let-7 over-expression decreased IL-10, as the silencing of let-7 miRNA led to a significant increase in IL-10 levels. HIV-1-infected HUT78 cells showed lower let-7 levels, accompanied by increased IL-10 levels, suggesting that the decreased let-7 level may be involved in the increased IL-10 expression seen in HIV-1 infection. They also observed reduced let-7 levels in primary CD4^+^ T-cells retrieved from the blood samples of subjects with HIV-1 infection compared with non-infected controls, suggesting that the altered miRNA levels could be linked to increased IL-10 expression in HIV patients. They proposed that dysregulation of the let-7/IL-10 axis could result in the abnormal CTL function seen in HIV-1-infected individuals [335]. Zhang et al. [336] reported that let-7i induced gene expression in Th-cells by binding to the TATA-box of the IL-2 promoter, thus promoting the assembly of pre-initiation complexes, which are required for mRNA transcription. They observed that HIV-1 infection resulted in lower levels of mature let-7i, as well as its precursor and primary forms. Additionally, studies have shown that the function of the let-7i promoter is reduced in Th-cells following HIV-1 infection. As a result, they suggest that viral infection results in the suppression of the let-7i/IL-2 axis, contributing to Th-cell death. This is a newly described mechanism for HIV-1-induced Th-cell death, where IL-2 cytokine can enhance the survival of activated T-cells [336].

#### 2.11.5. Neurotropic Viruses

Let-7a/b may play roles in the pathogenesis of the disease caused by JEV. The studies carried out have suggested that, in microglial cells, let-7a and let-7b can activate Notch in an NF-κB-dependent manner and through the TLR7-mediated signaling pathway, inducing the production of inflammatory cytokines such as TNFα. On the other hand, the extracellular release of let-7a/b via exosomes can transfer to neurons, promoting a neurotoxic effect and neuronal damage through the activation of cPARP and caspases-3/7/9 [337].

In summary, it is possible to state that the most important role of the let-7 family involves regulation of the immune response. It has been shown that the let-7 family can reduce the expression of pro-inflammatory cytokines and promote increased expression of the anti-inflammatory cytokine (IL-10) in most of the infections discussed in our review, thus preventing an excessive inflammatory response during viral infections. Researchers have also stressed that members of the let-7 family may affect viral replication.

### 2.12. MicroRNA-10a (miR-10a)

MiR-10a may be related to changes in immune homeostasis, and is down-regulated by many factors, such as TNF-α, IL-1β, and IL-6, as well as through promoting the production of the transcription factor YY1 [294,338]. The down-regulation of miR-10a accelerates inhibitor κB (IκB) degradation and NF-κB activation [294]. Furthermore, the reduced expression of miR-10a-5p leads to an increase in membrane-bound IL-6R [338]. Additionally, miR-10a inhibits DC expression of IL-12/IL-23p40 and NOD2, as well as inhibiting Th1 and Th17 cell functions [294]. 

#### 2.12.1. Hepatitis Viruses

Tan et al. [339] have observed elevated levels of miR-10a in the sera of patients with CHB. They reported that miR-10a may provide high diagnostic accuracy for CHB patients presenting persistently normal ALT with significant histological features and with no significant histological features. Additionally, miR-10a may be used to differentiate between the two units [339]. 

Furthermore, during HCV infection, the level of miR-10a is markedly up-regulated. Horii et al. [340] have observed that miR-10a regulates various liver metabolism genes and down-regulates the expression of the circadian rhythm gene brain and muscle aryl hydrocarbon receptor nuclear translocator-like 1 (Bmal1) by directly suppressing the expression of RAR receptor-related orphan receptor alpha (RORA) [340]. The over-expression of miR-10a in hepatocytes inhibited the expression of lipid synthesis gene sterol-regulatory element-binding protein (SREBP1), fatty acid synthase (FANS), SREBP2, gluconeogenesis-peroxisome proliferator-activated receptor gamma coactivator 1 alpha (PGC1α), protein synthesis mTOR and ribosomal protein S6 kinase (S6K), and bile acid synthesis liver receptor homolog 1 (LRH1). In addition, the down-regulation of Bmal1 by miR-10a was significantly correlated with the expression of mitochondrial biogenesis-related genes, increased serum ALT, and the progression of liver fibrosis in CHC. Therefore, miR-10a is a possibly useful biomarker for estimating prognoses in liver cirrhosis. Additionally, miR-10a is significantly associated with hepatitis C-related hepatocellular carcinoma recurrence. It has been suggested that miR-10a, through Bmal1, disturbs metabolic adaptations and leads to liver damage [340].

#### 2.12.2. Respiratory Viruses

Increased levels of miR-10a expression have also been observed during RSV infection. Zhang et al. [205] showed that hsa-miR-10a-3p was elevated in children with RSV-associated pneumonia. In addition, it was reported that, in children with severe RSV-associated pneumonia, the level of miR-10 was higher than that in children with mild RSV-associated pneumonia. These results indicate that hsa-miR-10a-3p may reflect severe RSV-associated pneumonia, and may serve as a potential candidate biomarker for severe RSV-associated pneumonia [205]. GO analysis indicated that most target genes for miRNAs, including miR-10a-3p, were involved in the NF-κB and MAPK signaling pathways, crucial components of many immune response processes in humans [205,341,342]. Furthermore, NF-κB is an important anti-apoptotic transcription factor for immune cells such as neutrophils, and plays an important role in damage repair during infection and inflammation [343]. Thus, the over-activation of NF-κB signaling may result in severe complications during severe RSV infection; as such, factors regulating its activity, such as miR-10a, are important.

#### 2.12.3. Cardiotropic Viruses 

Researchers have indicated that the miR-10a duplex is detectable in the cardiac tissues of suckling Balb/c mice, and can significantly up-regulate the biosynthesis of Coxsackievirus B3 (CVB3). Further research showed that the miR-10a target was located in the nt6818–nt6941 sequence of the viral 3D coding region. Taken together, these data suggest that miR-10a* can positively modulate host gene expression, and may play a role in the pathogenesis of CVB3 infection [344].

The presented studies indicate that the most important activity of miR-10a is related to regulation of the immune response. However, it is worth noting the role of miR-10a as a potential biomarker reflecting the severity of diseases of viral etiology.

Table 1 lists all of the miRNAs important in human viral infections, along with their target genes, biological functions, and modulated pathways.

## 3. MicroRNA Signature in Animal Viral Infections

In the scope of this study, we distinguished 10 host miRNAs that are important from the point of view of their participation in viral infections in animals (Figure 2B).

### 3.1. MicroRNA-155 (miR-155)

#### 3.1.1. Hemorrhagic Viruses

**Figure 3:**
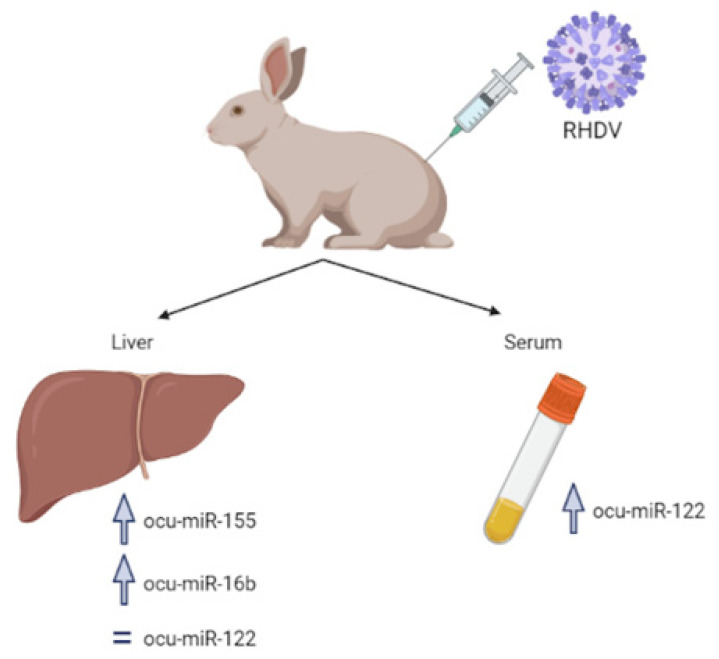
Signature of miRNAs ocu-miR-155-5p, ocu-miR-16b-5p, and ocu-miR-122-5p in the course of *Lagovirus europaeus*/RHDV infection [347].

Rabbit hemorrhagic disease virus (*Lagovirus europaeus*/RHDV) belongs to the family *Caliciviridae*, with an RNA genome. *Lagovirus europaeus*/RHDV is an etiological agent of rabbit hemorrhagic disease (RHD) [345], a highly infectious and deadly disease characterized by acute necrotizing hepatitis, but hemorrhages may also be found in other organs—in particular, the lungs, heart, kidneys, and spleen—due to disseminated intravascular coagulation [346]. The expression levels of ocu-miR-155-5p in liver were significantly higher (a fold change of 5.8) in infected rabbits, compared to the healthy rabbits (see Figure 3) [347]. The increased levels of miR-155 in liver tissues may be caused by the inflammatory responses in this organ caused by RHDV 12/24 h post-infection and the infiltration of cells of the immune system. Furthermore, a functional analysis has shown that ocu-miR-155-5p can regulate the expression of genes involved in processes related to acute liver failure (ALF) in rabbits. Furthermore, STRING analysis indicated the dependences between JUN, IGFR1, KRAS, TNF-α, TGF-β, IL-1β, IL-6, and IFN, as well as TLR, among others, in monocytes/macrophages and miR-155 [347]. These results indicate the role of miR-155 in both RHDV infection and the course of RHD, which may reflect the hepatic inflammation and impairment/dysfunction in RHD. The model used in this experiment (i.e., the infection of rabbits with *Lagovirus europaeus*/RHDV) is considered a good model for the study of viral hemorrhagic fevers in human, as well as multiple organ failure (MOF), as it exhibits biochemical and histopathological characteristics and clinical features that are remarkably similar to those in humans in the course of the disease [347].

#### 3.1.2. Respiratory Viruses

Avian influenza virus (AIV) belongs to the *Orthomyxoviridae* family of viruses, with an ssRNA genome [48]. AIV poses an ongoing risk to domestic pets, exotics, and wild birds worldwide. Disease outbreaks can threaten biodiversity due to their high morbidity and mortality in endangered species [48]. In AIV infection, researchers have shown a significant up-regulation of miR-155 [348]. Furthermore, based on target prediction, miR-155 could target the chicken anti-influenza gene MX dynamin-like GTPase 1 (MX1), as well as hemagglutinin (HA) and neuraminidase (NA), which are a major surface glycoproteins, therefore playing a role in host–AIV interactions in chickens. Additionally, up-regulated miR-155 might also activate the JNK pathway and, subsequently, induce apoptosis to eliminate virus-infected cells [348].

#### 3.1.3. Lymphotropic Viruses

Marek’s disease virus (MDV) is a member of the *Herpesviridae* family, with a dsDNA genome [349]. MDV, as a lymphotropic alphaherpesvirus of chickens, causes a disease characterized by tumor formation, immunosuppression, and neurological disorders [350,351]. With the emergence of new virulent strains in the field over time, MDV remains a serious threat to the poultry industry [350,351]. In deep sequencing studies of MDV-induced splenic tumors, Burnside et al. [350] reported decreased levels of miR-155, compared to normal spleen, resting T-cells, or activated T-cells [350]. These observations were confirmed by the in vitro study of Yao et al. [352], who observed a down-regulation of miR-155 specific to MDV-transformed tumor cells. In addition, the level of miR-155 expression was consistently reduced in all tested MDV-1-transformed lymphoblastoid cell lines, demonstrating that the down-regulation of miR-155 is a feature unique to the MDV transformation of T-cells. However, the molecular mechanisms that drives down the expression of miR-155 in MDV-transformed cell lines remains unknown [352]. 

In summary, it can be concluded that, in animals, the most important role of miR-155 is regulating the anti-viral response and reducing infection by inducing the apoptotic pathway in infected cells.

### 3.2. MicroRNA-223 (miR-223)

#### 3.2.1. Respiratory Viruses

AIV sub-type H1N2 (A/H1N2) is a sub-type of the influenza A virus. It is currently endemic in the pig population and is occasionally seen in humans. The virus does not cause more severe illness than other influenza viruses, and no unusual increases in influenza activity have been associated with it. In the research of Skovgaard et al. [353], it was shown that, in the lung tissue of pigs infected with A/H1N2, the expression levels of several miRNAs were increased, including miR-223, as observed one day after infection, followed by a decrease. This result suggests that miR-223 may be involved in controlling acute influenza infection in pigs [353].

#### 3.2.2. Lymphotropic Viruses

In MDV infection, miR-223 has been found to be down-regulated in all MDV-transformed cell lines relative to the levels in normal splenocytes or CD4+ T-cells. In addition, miR-223 was decreased in retrovirus-transformed AVOL-1 cells. Although the regulatory mechanisms are not yet fully understood, recent studies have indicated a clear role for miR-223 in hematopoiesis, as well as in malignancies, suggesting that miR-223 is involved in MDV-induced lymphocyte transformation [352].

#### 3.2.3. Vector-Borne Viruses

Vesicular stomatitis virus (VSV) is a member of the *Rhabdoviridae* family, with an ssRNA genome [285]. VSV is an arthropod-borne virus that primarily affects rodents, cattle, swine, and horses, but may also infect humans and other species. VSV infection occurs primarily in domesticated cattle, horses, swine, and rarely in llamas and humans, and can cause vesiculation, epithelial cell lysis, and severe interstitial edema, which appear with the infiltration of inflammatory cells [285]. MiR-223 has a key role in the development and homeostasis of the immune system, and plays an important role in VSV infection. A study showed that VSV induced the up-regulation of miR-223 in murine macrophages. Moreover, it was found that miR-223 over-expression up-regulated IFN-1 expression levels in VSV-infected macrophages and directly targeted FOXO3 to regulate IFN-1 production. This comprises the positive feedback regulatory function of miR-223 for the regulation of IFN-1 production in VSV infection [354]. 

The presented studies indicate that the most important activity of miR-223 in the considered viral infections is related to the control of viral infections and the regulation of factors involved in the anti-viral response.

### 3.3. MicroRNA-146a (miR-146a)

#### 3.3.1. Respiratory Viruses

Influenza affects the level of miR-146a in humans, but studies have indicated that influenza may also regulate this miRNA in another species [348,353,355]. AIV, outbreaks of which are worldwide threats to both poultry and humans, causes an infection of the respiratory tract, thus triggering a cascade of innate and adaptive immune responses and contributing to the up-regulation of miR-146a in chickens [348,355,356]. However, in the lung tissue of pigs infected experimentally with influenza virus (H1N2), the level of miR-146 was significantly decreased the first day after infection, which may affect the expression of IRAK1, STAT1, and TLR2 [353].

Hendra virus (HeV) is a member of the family *Paramyxoviridae*, with an RNA genome [357], and is a relatively emerging pathogen. It was first identified as the causative agent in an outbreak of severe respiratory disease and subsequent neurological disorders in horses. In addition, HeV may cause infection in humans, with high mortality rates [358]. MiR-146a is also induced in HeV infection; in particular, miR-146a has been found to be elevated in vitro in the blood of ferrets and horses infected with HeV, as well as in human cells [359]. Stewart et al. [359] have noted that induction of miR-146a during infection may be due to RIG-I. Additionally, studies have shown that inhibition of miR-146a reduces HeV replication in vitro. This suggests a role of this miRNA in HeV replication. This effect of miR-146a is mostly mediated through its target, ring finger protein 11 (RFN11), a member of the A20 ubiquitin-editing complex that negatively regulates NF-κB activity [359]. In another study, Cowled et al. [360] showed that HeV infection altered the expression profile of many host miRNAs, including miR-146a, as demonstrated by testing whole-blood samples during infection. The authors emphasized that the development of an miRNA signature in HeV infection would provide a chance for the early identification of infected horses, ensuring the possibility of reducing human exposure to infectious secretions and, thus, reducing the risk of transmission of zoonotic infection [360].

#### 3.3.2. Lymphotropic Viruses

The down-regulation of miR-146a has also been observed in MDV-induced splenic tumors [350]. MDV is a oncogenic virus caused by Marek’s disease (MD), which is a lymphoproliferative disorder in which aggressive T-cell lymphomas develop within two to six weeks following the infection of susceptible chickens [361].

#### 3.3.3. Vector-Borne Viruses

It was also found that VSV infection up-regulated miR-146a expression. MiR-146a was induced in mouse macrophages in a TLR–myeloid differentiation factor 88-independent but RIG-I/NF-κB-dependent manner during VSV. In turn, miR-146a negatively regulated VSV-triggered IFN-1 production, thus promoting VSV replication in macrophages. On the other hand, IRAK1 and IRAK2, other targets of miR-146a, also participate in VSV-induced IFN-1 production by associating with the Fas-associated death domain protein, an important adaptor in RIG-I signaling, in a VSV infection-inducible manner [116]. 

#### 3.3.4. Other Viruses

Foot-and-mouth disease virus (FMDV) is a member of the family *Picornaviridae*, with an RNA genome [362,363,364]. FMDV is the etiological agent of foot-and-mouth disease (FMD), known as one of the most contagious animal diseases. FMD targets both domestic and wild cloven-hoofed animals, and outbreaks have affected important livestock populations [363,364]. A study has shown that, during FMDV infection, one of dysregulated host miRNAs is miR-146a [365]. Stenfeldt et al. [365] have indicated that miR-146a is up-regulated in acute infection, which may modulate the immune response through induced NF-κB signaling by the targets IRAK1 and TRAF6, thus diminishing the pro-inflammatory response from TLR signaling [114,365,366]. 

Porcine Reproductive and Respiratory Syndrome Virus (PRRSV) is a member of the family *Arteriviridae*, with an RNA genome [367], and is characterized by episodes of reproductive failure in pregnant sows and respiratory illness, particularly in young pigs [368]. PRRSV infection contributes to the up-regulation of miR-146a in macrophages. An analysis has shown that miR-146a during PRRSV infection regulates tumor necrosis factor related protein 3 (C1QTNF3) and v-maf musculoaponeurotic fibrosarcoma oncogene homolog B (MAFB) genes, the decrease in which may aid in the immune response against PRRSV infection [369]. 

Our review indicates that, as in humans, miR-146a plays the most important role in the regulation of genes, influencing NF-κB activity and IFN production in viral infections in animals, making it an important factor in the anti-viral response.

### 3.4. MicroRNA-145 (miR-145)

MiR-145 is a molecule that has not been studied in humans in the course of viral infections so far. Additionally, there is very limited information about the function of miR-145 in the immune response. The only information that can be found regarding miR-145 is its function as a tumor suppressor, expressed in various tumors. However, recent research has focused on the role of miR-145 in various important animal viral infections [370]. 

#### 3.4.1. Respiratory Viruses

A decreased level of miR-145 was observed during swine-origin influenza A (H1N1) virus infection. Additionally, it was supposed that miR-145 targets the HA gene, which has been shown to be critical for the pathogenicity of influenza virus and immunosuppression. The authors suggested that miRNA-mediated host–virus interactions may characterize the location specificity of viral replication. Further studies focusing on these interactions are needed in order to reveal the interplay mechanism between the host miRNA and viruses [371]. 

#### 3.4.2. Neurotropic Viruses

Rabies virus (RABV) belongs to the *Rhabdoviridae* family, with an RNA genome, and is the etiological agent of rabies. Rabies is most often transmitted through being bitten by a rabid animal. RABV infects the central nervous system of mammals, ultimately causing disease in the brain and death [372]. Zhao et al. [373] observed a decrease in the level of miR-145 in the brain of mice infected with RABV. Informatic analysis indicated that miR-145 may play a role in the Jak–STAT signaling pathway (MYC; signal transducing adaptor molecule, STAM; STAT4; and SOCS7), cytokine–cytokine receptor interactions (IL17RB and TGFBR2), Fc gamma R-mediated phagocytosis (ADP ribosylation factor 6, ARF6; cofilin 2, CFL2; linker for activation of T-cells, LAT; actin related protein 2/3 complex subunit 5, ARPC5; and CT10 regulator of kinase-like Proto-Oncogene adaptor protein, CRKL), the Wnt signaling pathway (protein phosphatase 3 catalytic subunit alpha, PPP3CA; frizzled class receptor 9, FZD9; MYC; catenin beta interacting protein 1, CTNNBIP1; SMAD3; SUMO specific peptidase 2, SENP2; and WNT5B), or the TGF-beta signaling pathway (MYC; SMAD3; inhibin beta B, INHBB; TGFBR2; and SMAD5) [373]. 

In related studies, miR-145 has been considered only with respect to a few viruses, which makes it impossible to provide a final comment on the specific role of this miRNA in viral infections. It can only be suggested that miR-145 is down-regulated by viral infections and may participate in various signaling pathways related to the immune system.

### 3.5. MicroRNA-21 (miR-21)

#### 3.5.1. Respiratory Viruses

During influenza virus infection in pigs and chickens, the up-regulation of miR-21 has been observed. According to the authors of several studies, miR-21 has a very important function in lymphocyte development and modulations, and may also regulate the expression of CXCL10, which is a chemoattractant for monocytes/macrophages, T-cells, and NK cells. The results of their studies demonstrate that miR-21 is an innate host immune factor, and participates in the response to viral infection [353,355,374]. 

#### 3.5.2. Neurotropic Viruses

A study has shown that miR-21 can be expressed differently throughout RABV infection, in a time-dependent manner. The greatest increase in miR-21 in cultured murine RABV-infected neurons occurred at 144 h of infection, reaching a fold change of about 4.0, while it reached a fold change of about 2.0 in hippocampal neurons. The results indicated the participation of this miRNA in neuronal dysfunction caused by RABV infection. However, more research is needed to elucidate the role of miR-21 in RABV infection [375].

In conclusion, it should be noted that miR-21, in the above viral infections, on one hand, participates in the immune response against viral infections; on the other hand, it also potentially affects the dysfunction of the infected organs.

### 3.6. MicroRNA-16/miR-15a Cluster (miR-16/miR-15a Cluster)

MiR-16/miR-15a inhibits cell proliferation through the regulation of a number of proliferation-related targets and apoptosis [376]. Similarly to miR-145 and miR-21, very limited research has been carried out regarding the miR-15a/miR-16 cluster in viral infections.

#### 3.6.1. Hemorrhagic Viruses

During infection with *Lagovirus europaeus*/RHDV, a significantly higher level of ocu-miR-16b-5p has been observed in the liver of infected rabbits compared to healthy rabbits (a fold change of 2.5) see Figure 3. Therefore, it has been suggested that miR-16b may play a role in the pathogenesis of RHD. Due to its role, ocu-miR-16b-5p may promote liver cell apoptosis by targeting Bcl-2 in response to viral infection, but is also crucial for an effective inflammatory response in damaged liver tissue. In addition, miR-16, by regulating HGF, is required for cell proliferation during the liver regeneration process [347].

#### 3.6.2. Respiratory Viruses

MiR-15a has been found to be significantly up-regulated after influenza A infection in lung tissues at all time points (i.e., 1 day, 3 days, and 14 days post-infection). However, target genes of miR-15a were also increased (moderately for TLR7 and strongly for CXCL10). These results indicate that miR-15a may not be involved in the observed regulation of TLR7 and CXCL10 mRNA levels, or that this miRNA acts through the repression of mRNA translation to protein, although no data yet support this notion [353]. 

In summary, it should be stated that the most important role of the miR-15a/miR-16 cluster concerns, on one hand, its participation in the immune response and apoptosis, and, on the other hand, organ regeneration in the course of viral infections.

### 3.7. MicroRNA-181 Family (miR-181 Family)

The miR-181 family consists of members involved in the regulation of many relevant biological processes, including cell proliferation, apoptosis, autophagy, mitochondrial function, and immune responses [377]. 

#### 3.7.1. Respiratory Viruses

Low expression levels of miR-181a and miR-181b have been observed in avian influenza infection. The author suggested that miRNAs with different expression levels are important in the immune response to viral infection. The above miRNAs are involved in cell proliferation, apoptosis, and other biological processes; however, further studies are required to better understand the prevalence and functions of miR-181 family members in animal viral infections [378]. 

#### 3.7.2. Lymphotropic Viruses

In MDV infection, apart from miR-181b, another member—miR-181a—has also presented a lower level of expression in splenic tumors. The research suggested that the miR-181 family is very important to this virus, playing roles in immune response evasion [350]. 

#### 3.7.3. Other Viruses

During persistent infection with FMDV, the down-regulation of miR-181b in cow sera has been observed. MiR-181b may regulate cellular proliferation by targeting RASSF1A and NF-κB, and can also exert an immunomodulatory effect through adenylyl cyclase 9 (AC9) and inhibition of IFNα expression. However, decreased expression of miR-181b in FMDV infection may affect the increased production of IFNα, thus leading to a better anti-viral response [365]. 

However, in PRRSV infection, only miR-181c presented a decreased expression level in porcine alveolar macrophages (PAMs). Meanwhile, an in vitro study has indicated that both miR-181a and miR-181c inhibit viral gene expression and PRRSV production by specifically binding to a highly conserved region downstream of ORF7 in the viral genomic RNA [5]. Additionally, miR-181c can down-regulate the PRRSV receptor CD163 in blood monocytes and PAMs. Decreased CD163 levels lead to the inhibition of PRRSV entry into PAMs, subsequently suppressing PRRSV infection [379].

In summary, it can be concluded that miR-181, in the considered viral infections in animals, participates in regulation of the immune response.

### 3.8. Let-7 Family (Let-7)

#### 3.8.1. Lymphotropic Viruses

In vitro research conducted by Tian et al. [380] has shown that the expression of let-7i is down-regulated in chicken cell lines during MDV infection. They also found that, similarly to the infected line, let-7i was decreased in MD tumors in infected chickens. Further bioinformatic analyses showed that let-7i may interact with activating transcription factor 2 (ATF2) mRNA in its coding regions, which regulates proliferation and apoptosis, and might (along with another miRNAs) be related to Marek’s disease resistance/susceptibility [380]. 

Avian leukosis virus (ALV) belongs to the *Retroviridae* family, with an RNA genome. ALV induces myeloid leukosis (ML) primarily in broilers, leading to major economic losses in the poultry industry worldwide [381]. During ALV infection, let-7b and let-7i presented variable expression, over 120 days of infection, in liver and bone marrow tissues [382]. Meanwhile, Li et al. [383] reported only down-regulation of the tested miRNAs [383]. Through use of bioinformatic analysis, it was predicted that both gga-let-7b and gga-let-7i are involved in multiple pathways, including signaling pathways based on factors such as MAPK, TGF-β, Notch, Wnt, mTOR, cell cycle, P53, and Jak-STAT. Based on these data, the researchers suggested that let-7b and let-7i likely play critical roles in regulating tumorigenesis in the course of AVL infection [382]. 

#### 3.8.2. Other Viruses

Stenfeldt et al. [365] paid attention to the role of let-7g in the course of acute FMDV infection, and showed that the level of let-7g was down-regulated twice in acute infection. Furthermore, they indicated the role of let-7g in cellular proliferation by targeting lectin-like oxidized low density lipoprotein receptor-1 (LOX) or caspase-3. Additionally, it was observed that, after the treatment of FMDV infection, let-7g levels returned to the baseline levels before infection, supporting the use of serum miRNA profiling to identify infected FMDV carriers [365].

The presented studies indicate that the most important activity of the let-7 family in the considered viral infections is related to the proliferation and apoptosis of cells.

### 3.9. MicroRNA-122 (miR-122)

#### 3.9.1. Hemorrhagic Viruses

Results for ocu-miR-122-5p were obtained in rabbits infected with *Lagovirus europaeus*/RHDV (see Figure 3), from which it was observed that the level of miR-122 was not significantly different in the liver tissue of infected rabbits compared to healthy rabbits. However, in sera from RHDV-infected rabbits, the miR-122 level was higher compared to that from controls [347]. Additionally, the results of a GO analysis for ocu-miR-122-5p indicated that its potential role in the response to RHDV infection includes regulation of the expression of genes involved in the processes of hepatic homeostasis (e.g., HGF and c-Met) and, to a lesser extent, apoptosis (e.g., STAT1 and STAT3). Based on these data, the researchers suggested that ocu-miR-122-5p in serum may potentially serve as a biomarker of liver damage from RHD [347].

#### 3.9.2. Respiratory Viruses

An increase in miR-122 has been observed in the lungs of chickens in the course of AIV infection. A comprehensive analysis, combining both gga-miR-122–1 and 122–2 with targeted mRNA gene expression for MX1, IL-8, IRF-7, and TNFRS19, indicated that they are strong candidate miRNAs and genes involved in regulation of the host response to AIV infection in the lungs of broiler chickens. Further miRNA- or gene-specific knock-down assays are thus warranted in order to elucidate the underlying mechanism of AIV infection regulation in chickens [348]. 

Our review indicates that the role of miR-122 in viral infections in animals may vary, depending on the tissue environment. On one hand, it can influence liver homeostasis; on the other hand, it affects the immune response in infected organs. In addition, researchers have noted the role of miR-122 in animals as a potential marker of liver damage in the course of viral infection.

Table 2 lists all of the miRNAs that are important in animal viral infections, along with their target genes, biological functions, and modulated pathways.

## 4. Conclusions

MiRNAs are non-coding RNAs that play key roles under pathological conditions in humans and animals, including viral infections. In this review, we summarized the available literature data, indicating that the signature miRNAs in human viral infections mainly include 12 miRNAs (i.e., miR-155, miR-223, miR-146a, miR-122, miR-125b, miR-132, miR-34a, miR -21, miR-16, miR-181 family, let-7 family, and miR-10a), while 10 key miRNAs were determined in animals (i.e., miR-155, miR-223, miR-146a, miR-145, miR-21, miR-15a/miR-16 cluster, miR-181 family, let-7 family, and miR-122). 

The biological potential of host miRNA during viral infections is due, on one hand, to the fact that they can act as anti-viral microRNAs, inhibiting viral replication; on the other hand, they may also act as pro-viral microRNAs, necessary for the virus to replicate in the cellular environment. In addition, host microRNAs are essential regulators of the response to viral infections. Knowledge of host miRNAs and their roles in various viral infections can provide a useful tool for identifying the biological functions of genes and pathways that are activated in order to elicit an effective immune response, and other biological processes involved in the anti-viral response, such as apoptosis and oxidative stress.

Changes in miRNA expression during viral infections precede the detection of the viral genome in the studied material, confirming the view that determination of the miRNA signature can be used for the early diagnosis of many viral diseases, including Hendra virus infection of horses; however, the development of miRNAs as disease-specific biomarkers likely requires more comprehensive measurement and the determination of very detailed miRNA signatures.

Knowledge of which miRNAs are involved in different viral infections and the biological roles that they play can help in understanding the pathogenesis of viral diseases, facilitating the future development of therapeutic agents for both humans and animals.

## Figures and Tables

**Table 1:** MicroRNAs important in human viral infections.

MicroRNA	Virus/Disease	Target Genes	Biological Function/Modified Pathways	Reference
miR-155	HBV/HB	SOCS1	Enhances the phosphorylation of STAT1 and STAT3; enhances JAK/STAT signaling pathway; suppresses HBV infection in hepatocytes; regulates IFN-γ production; inhibits Akt/mTOR pathway	[23,26,29]
		C/EBP-β	Modulates HBV replication	[24]
		BCL-6, SHIP-1, SOCS-1	Induces inflammatory cytokine production	[28]
	HCV/HC	TNF-α	Pro-inflammatory response	[33]
		Tim3/T-bet	Increases IFN-γ production; regulates Tim-3/T-bet/STAT-5 signaling and cytokine expression in NK cells; immune injury during chronic viral disease	[36]
		ISG15, TLR3	Innate and adaptive immune response	[34]
		APC	Wnt pathway; apoptosis; cell proliferation	[39]
	DENV/dengue	Bach1	Replication of DEVN; enhances anti-viral IFN responses	[43]
		SOCS1	Regulates cytokine signal transduction	[41]
	WNV/ West Nile fever	IL-13, BDNF, CCR9	Cell survival	[45]
		IL-1β, IL-12, IL-6, IL-15, GM-CSF	Anti-viral response	[47]
	IAV/influenza	S1PR1	Inflammatory response; activates S1PR1/NF-κB/pro-inflammatory cytokine pathway	[51]
		IFN type I	Anti-viral response	[53]
	RSV/upper respiratory tract infections	IFN type I	Anti-viral response	[53]
		TNF-α, IL-1β, IL-6, IL-8	Pro-inflammatory response	[59]
		SHIP1, Kif1	Antigen presentation	[61,62]
		SOCS1	Enhances activation of STAT1 and up-regulation of ISGs gene	[63]
	SARS-CoV-2/ COVID-19	STAT1, STAT3, TGFB1, SMAD3, IRF1, AKT1, MYB, BCL6, TP6, HIF1A, FOXP3, JUNB, NFKB1	Immune response; apoptosis	[71]
		SOCS1, IL-6, IL-1β, CSF1R CD274, TLF6, TNF	Regulates the host immune response; miR-155-5p–IL-6/TNF/IL-1β axis	[75]
		IL-1α, G-CSF, IL-9, MIP-2, IL-12-P70 VEGF, IP-10, IFN-γ, MCP-1, MIG, MIP-1α, M−CSF, TNF-α, MIP-1β	Alleviates inflammation and lung cytokine storm	[73]
	HAdV/respiratory infections (colds)	IFN-β	Anti-viral response	[80,81]
		SOCS1	Enhances type I IFN Anti-viral response	[82]
		SHIP1	Enhances IFN type I signaling	[20]
	HRV/COPD	HRV1B	Inhibits viral replication	[84]
miR-223	HCV/CHC	NF-κB	Chronic liver inflammation	[94]
	DENV/dengue	STMN1	DENV replication	[95]
	IAV/influenza	TNF-α	Pro-inflammatory response	[97,101]
		PI3K, IGF1R, GPCR, PP2A, PKA and Ca2+ channel	Represses the activity of CREB; T-cell development and cell survival	[96]
		IL1RN, MDA5, STAT1	Cell death; apoptosis	[100]
	SARS-CoV/SARS	NF-κB	Activated CCR1, the inflammatory chemokine receptor for CCL3 and CCL5 enhances lung fibrosis	[105]
	SARS-CoV-2/ COVID-19	TRAF6, FOXO1, TLR4, STMN1, PI3K/AKT, CXCL2, CCL3, IL-6, IFN-I, IL-1β, Caspase-1 and mainly NLRP3, IKKα, NF-κB	Regulates inflammatory processes; anti-oxidant and anti-viral role	[108]
	HIV-1/AIDS	RhoB Sp3, LIF	Inhibits HIV-1 production in resting primary CD4+ T-cells Activates the AKT–NF-κB pathway Viral replication	[110] [112] [112]
miR-146a	HBV/CHB	NF-κB	Production of pro-inflammatory cytokines (TNF-α, IL-6, IL-8, IL-12, and IL-18)	[118]
		STAT1	Viral persistence Decreases cytotoxicity of T-cells	[117]
		CFH	Regulation of the complement alternative pathway	[121]
		XIAP	Regulation of HBV replication; regulation of XIAP-MDM2/p53 pathway	[118]
		ZEB2	Regulation of HBV transcription and replication	[119]
		TRAF6, IRAK1	Regulation of FEN1 by NF-κB activity; promotes HBV DNA replication	[120,124]
	HCV/HC	SOCS1	STAT3 inhibition; increases IL-23, IL-10, and TGF-β expression	[125]
	DENV/dengue	TRAF6 LC3	Inhibits IFN-β and IL-28A/B; increases DENV2 replication Autophagy; viral replication	[129] [130]
	IAV/influenza	TRAF6	Production of type I IFN; anti-viral response	[133]
		IRAK1	IL-7, VEGF and, JAK-STAT signaling pathway	[134]
	RSV/upper respiratory tract infections	TRAF6, IRAK1	Viral replication	[137,138]
	SARS-CoV-2/ COVID-19	TRAF6, IRAK1, IRAK2	NF-κB pro-inflammatory signaling pathway	[139]
		STAT1	Regulates JAK/STAT pathway	[139]
miR-122	HBV/HB	cyclin G1	Replication of HBV	[149]
		HO-1	Replication of HBV; oxidative stress	[153,154,155]
		IFN type 1	Replication of HBV; anti-viral response	[156]
		SOCS3	JAK/STAT pathway signaling; cytokine signaling	[157]
	HCV/HC	viral 5′-NCR	Replication of HCV	[166]
		Bach1	HO-1 gene regulation; HCV replication	[170]
		IFN	Anti-viral response	[171]
		TGFBRAP1	Promotes HCC progression induced by HCV	[172]
	DENV/dengue	CYP7A1, IGFR1, SFR, RAC1, RHOA, cyclin G1	Immune response	[131]
	ZEBOV/RESTV/ Ebola	viral vp40 gene	Regulation of virus replication	[176,177]
	RSV/upper respiratory tract infections	Wnt	Inflammatory and immune response	[178]
		IL1R1	NF-κB activation, inflammatory response	[179,180]
		TLR4	Innate immune response: NF-κB activation; cytokine secretion; inflammatory response	[180]
		iNOS	Anti-viral response	[184]
	HRV/COPD	CXCL2	Chemotaxis	[185]
		SOCS1	Regulates cytokine signal transduction	[185]
miR-125b	HBV/HB	LIN28B	Regulates let-7 and stimulates HBV replication	[194]
		SCNN1A	Inhibits HBV core protein expression	[197]
	HCV/HC	TLR2	TLR2/MyD88 signaling pathway; phosphorylation of NF-κBp65, ERK, and P38	[198]
		HuR	Viral replication	[200]
	RSV/upper respiratory tract infections	NF-κB, MAPK	Immune response	[205]
	IAV/influenza	MAPK	Cell proliferation; apoptosis	[206]
	SARS-CoV-2/ COVID-19	ACE2	Activation of immune system	[207]
	HIV-1/AIDS	CPSF6	Regulation of HIV-1 nuclear entry and viral replication	[208]
	JEV/Japanese encephalitis	STAT3, Map2k7, Triap1	Reduces genome replication and virus titers	[212]
miR-132	HBV/HB	Akt	Development of HCC induced by HBV	[217]
	IAV/influenza	IRF1	Regulation of IFN-α and IFN-β production	[219]
		MAPK3	Regulates innate immune signaling pathways	[134]
	HSV/orofacial and genital disease	Ras	Inflammation	[224]
miR-34a	HBV/HB	Wnt1	Regulation of Wnt/β-catenin pathway; contributes to HCC induced by HBV	[229]
		HMGB1	Innate immune response; apoptosis	[227]
		CCL22	Chemotactic for monocytes, dendritic cells, natural killer cells, and T lymphocytes	[230]
		Smad4	Regulation of TGF-β/Smad3 pathway activity; liver fibrosis	[228]
	DENV/dengue WNV/West Nile fever	Wnt	Activates type I IFN response; regulation of WNT/β-catenin signaling; anti-viral state	[233]
	IAV/influenza	Bax	Apoptosis	[234]
		STAT3	Anti-inflammatory function Inhibits NF-κB gene reporters	[235]
	SARS-CoV-2/ COVID-19	Bcl-2, Bax, KLF4 IL-6R	Apoptosis Inflammation	[236]
	HTLV-1/leukemia	SIRT1 Bax	Apoptosis	[240]
miR-21	HBV/CHB	PTEN	PTEN/Akt signaling; activation of HSCs; apoptosis	[250,251]
		Smad7	Promotes α-SMA and collagen I expression in HSCs; liver fibrosis	[250,251]
		TGF-β1	Positive feedback loop; liver fibrosis	[249]
		PDCD4	Apoptosis	[252]
		IL-12	Regulates proliferation of NK and T-cells	[257]
	HCV/HC	PTEN	Apoptosis	[260]
		MyD88 IRAK1	Production of IFN type I; anti-viral response	[261]
		Smad7	Fibrosis	[262]
	IAV/influenza	CCL1, CCL17, CCL19, IL-22, C2orf28	Inflammation; apoptosis	[268]
		TGF-β	Fibrosis	[271]
	Ad/respiratory infection	p53 TGF-β	Apoptosis	[80]
	SARS-CoV-2/ COVID-19	ORF1ab ORF3a S protein gene	Regulation of viral entry	[273]
		CCL20	Chemotactic response TGF-β and Akt signaling	[139]
		IRAK1	Participates in NF-κB pro-inflammatory signaling pathway	[139,273]
		CXCL-10	Pro-inflammatory response; chemotaxis	[273]
	HIV-1/AIDS	PTEN PDCD4 CDKN1B	Apoptosis	[279]
		IP-10	Inflammation	[280]
	CVB3	YOD1	Metabolism of proteins; protein ubiquitination	[284]
	CHPV	PTEN	Regulation of phosphorylation of AKT and subunit p65 of NF-ĸB; production of pro-inflammatory cytokines (IL-6, TNF-α)	[286]
miR-16	HBV/HB	CCND1	Cell proliferation	[290]
		c-Myc	Cellular metabolism and proliferation	[290]
		Bcl-2	Apoptosis	[290]
	HCV/HC	HGF	Hepatocyte proliferation	[291]
		Smad7	Fibrosis	[291]
	Human CoV (MERS, SARS, SARS-CoV-2)	polyprotein 1ab coding region	Regulation of viral replication	[272]
	SARS-CoV-2/ COVID-19	MK2 CCND1	Cell proliferation	[273]
	RSV/upper respiratory tract infections	TLR4 RIG-1	Activation of NF-κB; pro-inflammatory	[58]
miR-181a	HBV/HB	PTEN	Cell proliferation; apoptosis; development of hepatocellular carcinoma	[300]
		CLDN1	Blocks HCV entry into cells	[306]
		E2F5	Development of HCC induced by HBV	[301]
		Fas	Apoptosis and regulation of HCC cell proliferation	[302]
	HCV/CHC	DUSP6	Regulation of T-cell response	[305]
miR-181b	HBV/HB	p27 PTEN	Regulation of hepatic stellate cell proliferation; pro-fibrotic role	[298,299]
miR-181c	HCV/HC	HOXA1	Cell growth regulator; activates STAT3 and STAT5	[306]
		ATM	Apoptosis	[307]
		CDK-2 cyclin-A	Cell cycle	[307]
	IAV/influenza	Bcl-2	Apoptosis	[206]
		IL-2, TNF-α	Immune response	[206]
let-7a	HBV/HB	STAT3	Immune suppression	[315,317]
	HCV/HC	CHUK, IKBKE, XPNPEP1	Regulation of HCV assembly or secretion	[320]
	JEV/Japanese encephalitis	cPARP	Apoptosis	[337]
let-7b	HCV/HC	IFN type I SOCS1 IKKα	Immune response; anti-viral response; stimulation of JAK/STAT signaling pathway	[322]
		GF2BP1	Regulation of HCV replication	[323]
	SARS-CoV-2/ COVID-19	RELB, IL-6, KHSRP, EHMT2, KDM2B, CMYC, LIN28B	Immune response; repair function	[71]
		TLR4	Regulation of inflammation via NF-κB	[333]
		IL-6, IL-8, TNF-α, IL-10	Regulation of pro- and anti-inflammatory cytokine production	[333]
		ORF1ab (virus gene)	Regulation of viral replication	[238]
	HIV-1/AIDS	IL-10	Anti-viral response	[335]
	JEV/Japanese encephalitis	cPARP	Apoptosis	[337]
let-7c	DENV/dengue	Bach1	Regulation of HO-1; regulation of viral replication; oxidative stress; anti-inflammatory response	[326]
let-7f	RSV/upper respiratory tract infections	CCL7 SOCS3	Anti-viral cytokine response	[330]
let-7i	HIV-1/AIDS	IL-2	Th-cell death	[336]
miR-10a	HCV/HC	RORA	Immune response	[340]
		SREBP1 FANS SREBP2 PGC1α	Lipid homeostasis; cholesterol biosynthesis; fatty acid synthesis; regulation of fatty acid metabolism	[340]
		Bmal1	Liver damage	[340]
	RSV/upper respiratory tract infections	NF-κB MAPK	Immune response	[341,342,343]

The abbreviations in the table are explained in the text.

**Table 2:** MicroRNAs that are important in animal viral infections.

MicroRNA	Virus/Disease	Target Genes	Biological Function/Modified Pathways	Reference
miR-155	*Lagovirus europaeus*/RHDV/ RHD	JUN, IGFR1, KRAS, TNF-α, TGF-β, IL-1β, IL-6, IFN	Immune response	[347]
	AIV/influenza	MX1	Anti-viral response	[348]
		JNK pathway	Apoptosis	[348]
miR-223	VSV/vesicular stomatitis	FOXO3	Regulation of IFN type I production	[354]
miR-146a	H1N2/influenza	IRAK1 STAT2 TLR2	Innate immune response; regulation of IFN type I production	[353]
	HeV	RIG-I	Innate immune response	[359]
		RFN11	Regulation of viral replication	[359]
	VSV/vesicular stomatitis	IRAK1, IRAK2	Regulation of IFN type I production	[116]
	FMDV/FMD	TRAF6 IRAK1	Regulation of NF-κB activation; innate immune response	[365]
	PRRSV/PRRS	C1QTNF3 MAFB	Regulation of immune response	[369]
miR-145	AIV/influenza	HA	Regulation of pathogenicity of influenza virus and immunosuppression	[371]
	RABV/rabies	MYC, STAM, STAT4, SOCS7	JAK/STAT signaling pathway	[373]
		IL17RB TGFBR2	Receptor for the pro-inflammatory cytokines IL17B and IL17E, controlling the growth and/or differentiation of hematopoietic cells Regulation of cell cycle arrest in epithelial and hematopoietic cells; control of mesenchymal cell proliferation and differentiation; wound healing; extracellular matrix production; immunosuppression; and carcinogenesis	[373]
		MYC, SMAD3, INHBB, TGFBR2, SMAD5	TGF-beta signaling pathway	[373]
		ARF6, CFL2, LAT, ARPC5, CRKL	Fc gamma R-mediated phagocytosis	[373]
miR-21	H1N2/influenza	CXXL10	Chemoattractant for monocytes/macrophages, T-cells, NK cells, and dendritic cells; may also promote T-cell adhesion to endothelial cells	[353,355]
miR-16 miR-15a	*Lagovirus europaeus*/RHDV/RHD	HGF Bcl-2	Cell proliferation during the liver regeneration process Apoptosis	[347]
	AIV/influenza	CXCL10	Chemoattractant for monocytes/macrophages, T-cells, NK cells, and dendritic cells; may also promote T-cell adhesion to endothelial cells	[353]
		TLR7	Activation of innate immunity	[353]
miR-181a	PRRSV/PRRS	ORF7 (virus gene)	Inhibits viral gene expression and virus protein production	[379]
miR-181b	FMDV/FMD	RASSF1A NF-κB	Regulates cellular proliferation and inflammatory response	[365]
		AC9	Immunomodulatory effect	[365]
		IFNα	Anti-viral response	[365]
miR-181c	PRRSV/PRRS	ORF7 (virus gene)	Inhibits viral gene expression and virus protein production	[379]
let-7g	FMDV/FMD	LOX	Cell proliferation	[365]
		caspase-3	Apoptosis	[365]
let-7i	MDV/MD	ATF2	Proliferation, apoptosis	[380]
miR-122	*Lagovirus europaeus*/RHDV/RHD	HFG, c-Met STAT1, STAT3	Hepatic homeostasis Apoptosis	[347]
	AIV/influenza	MX1, IL-8, IRF-7, TNFRS19	Immune response	[348]

The abbreviations in the table are explained in the text.

## Data Availability

Not applicable.
